# Adolopment of adult diabetes mellitus management guidelines for a Pakistani context: Methodology and challenges

**DOI:** 10.3389/fendo.2022.1081361

**Published:** 2023-01-05

**Authors:** Russell Seth Martins, Muhammad Qamar Masood, Omar Mahmud, Nashia Ali Rizvi, Aisha Sheikh, Najmul Islam, Anum Naushad Ali Khowaja, Nanik Ram, Saira Furqan, Mohsin Ali Mustafa, Salima Saleem Aamdani, Alina Pervez, Adil H. Haider, Sarah Nadeem

**Affiliations:** ^1^ Center for Clinical Best Practices, Clinical and Translational Research Incubator (CITRIC), Aga Khan University, Karachi, Pakistan; ^2^ Section of Endocrinology, Department of Medicine, Aga Khan University, Karachi, Pakistan; ^3^ Medical College, Aga Khan University, Karachi, Pakistan; ^4^ Department of Medicine, Aga Khan University, Karachi, Pakistan

**Keywords:** Type 2 diabetes mellitus, screening, lower-middle-income country, adolopment, screening

## Abstract

**Introduction:**

Pakistan has the highest national prevalence of type 2 diabetes mellitus (T2DM) in the world. Most high-quality T2DM clinical practice guidelines (CPGs) used internationally originate from high-income countries in the West. Local T2DM CPGs in Pakistan are not backed by transparent methodologies. We aimed to produce comprehensive, high-quality CPGs for the management of adult DM in Pakistan.

**Methods:**

We employed the GRADE-ADOLOPMENT approach utilizing the T2DM CPG of the American Diabetes Association (ADA) Standards of Medical Care in Diabetes – 2021 as the source CPG. Recommendations from the source guideline were either adopted as is, excluded, or adapted according to our local context.

**Results:**

The source document contained 243 recommendations, 219 of which were adopted without change, 5 with minor changes, and 18 of which were excluded in the newly created Pakistani guidelines. One recommendation was adapted: the recommended age to begin screening all individuals for T2DM/pre-diabetes was lowered from 45 to 30 years, due to the higher prevalence of T2DM in younger Pakistanis. Exclusion of recommendations were primarily due to differences in the healthcare systems of Pakistan and the US, or the unavailability of certain drugs in Pakistan.

**Conclusion:**

A CPG for the management of T2DM in Pakistan was created. Our newly developed guideline recommends earlier screening for T2DM in Pakistan, primarily due to the higher prevalence of T2DM amongst younger individuals in Pakistan. Moreover, the systematic methodology used is a significant improvement on pre-existing T2DM CPGs in Pakistan. Once these evidence based CGPs are officially published, their nationwide uptake should be top priority. Our findings also highlight the need for rigorous expanded research exploring the effectiveness of earlier screening for T2DM in Pakistan.

## Introduction

Type 2 diabetes mellitus (T2DM) is amongst the top ten leading causes of death worldwide and continues to burden healthcare systems globally (1). Lower-middle-income countries (LMICs) bear the brunt of this disease, with almost 80% of patients with T2DM belonging to LMICs (2). In Pakistan, a South Asian LMIC with a population of over 220 million, the prevalence of T2DM amongst adults as reported by the International Diabetes Federation (IDF) is more than one in four adults (26.7%) (3). This is the highest national prevalence in the world. As a consequence, Pakistan currently has the third highest number of people living with T2DM in the world, behind only China and India (3), and is predicted to lead the list in the near future (4).

Evidence-based clinical practice guidelines (CPGs) are the cornerstone of the evaluation and management of T2DM. The majority of T2DM CPGs followed internationally have been designed by high-income countries in the West, such as the United States of America (USA) (5, 6), the United Kingdom (UK) (7), and Canada (8, 9). These CPGs have been developed taking into account the healthcare systems of their respective countries, from where, understandably, the majority of the most highly influential T2DM-related research originates (10). Furthermore, LMICs often lack the financial resources and research infrastructure to produce evidence-based CPGs locally and independently (11). This presents a problem, as many factors affecting the management of T2DM differ in LMICs like Pakistan, including epidemiology (3), surveillance systems (12), healthcare costs (13), complication rates (14), sociocultural influences (15), quality of life (16), disease-related awareness (17), health-seeking behavior (17), healthcare infrastructure (18), healthcare access (18), diet (19), and self-care (20). Recognizing this, it is also recommended that even internationally-used best-evidence CPGs need to be modified according to the healthcare context that plans to use them (8). This holds particularly true for LMICs, especially those in South Asia, which suffer from relatively greater burdens of disease.

*De novo* creation of CPGs is a laborious, resource-intensive process. When not possible due to lack of resources, as is the case in Pakistan and other LMICs, the optimal modification process should be based on a combination of adoption (assimilating existing recommendations as is), adaptation (modification of selected recommendations following critical evaluation), and exclusion (omitting recommendations deemed irrelevant to local context) of existing CPGs (11). Adolopment is a recently introduced word that encompasses three key elements of adoption, adaptation, and development (21).The GRADE (Grading of Recommendations, Assessment, Development and Evaluation)-ADOLOPMENT (11) process uses evidence to decision (ETD) tables to guide this process (22–24). ETD tables provide general and context-specific evidence across standard criteria (**Table 1**) against which experts judge the overall appropriateness of existing recommendations and proposed modifications. These modifications may be in the form of a change to the specific population, intervention, or control as compared to the original recommendation.

**Table 1:** Summary of Judgments.

Question: “Should we recommend screening in persons younger than 45 years vs. above 45 years be used for Diabetes/Prediabetes screening in Pakistan?”			
Criteria	Summary of Judgments: n/N (%)	Consensus Judgment	Additional Comments from Panel Discussion
**Problem**	• Yes: 5/5 (100%)	Yes	“This is a question that must be most highly prioritized by all those involved in the management of T2DM in Pakistan.”
**Desirable Effects**	• Moderate: 1/5 (20%) • Large: 4/5 (80%)	Large	“T2DM can often coexist with other debilitating conditions that serve as risk factors, such as polycystic ovarian syndrome (PCOS). Diagnosing T2DM earlier and beginning timely intervention will help improve quality of life pertaining to other co-existing conditions as well.”
**Undesirable Effects**	• Small: 4/5 (80%) • Trivial: 1/5 (20%)	Small	“We can all agree that there are no direct undesirable effects to the health of the patient if earlier screening is implemented.”
**Values**	• Possibly important uncertainty/variability: 2/5 (40%) • Probably no important uncertainty/variability: 3/5 (60%)	Probably no important uncertainty/variability	“While patients in Pakistan may not attribute due importance to matters considering screening and timely diagnosis of T2DM, this likely stems from a lack of knowledge and awareness rather than a lack of value.”
**Balance of Effects**	• Favors the Comparison: 1/5 (20%) • Probably favors the Intervention: 1/5 (20%) • Favors the Intervention: 3/5 (60%)	Favors the intervention	“Overall, while there is a lack of high-quality evidence regarding the benefits of lowering the age of screening for T2DM in our population, the risks or disadvantages are non-existent in comparison.”
**Resources Required**	• Moderate Costs: 1/5 (20%) • Negligible Costs and Savings: 1/5 (20%) • Moderate Savings: 3/5 (60%)	Moderate savings	“The resources required for T2DM screening are already in place in most healthcare testing facilities.”
**Certainty of Evidence of Required Resources**	• Low: 2/5 (40%) • Moderate: 3/5 (60%)	Moderate	“We were able to confirm that most major healthcare testing facilities, with branches across the country, offer T2DM testing.”
**Cost-Effectiveness**	• Favors the Comparison: 2/5 (40%) • Probably favors the Intervention: 1/5 (20%) • Favors the Intervention: 2/5 (40%)	Favors the intervention	“Despite the lack of evidence, we believe it is more cost-effective for the patient and healthcare system to engage in earlier screening than risking greater expenditure managing the complications of T2DM in the future”
**Equity**	• Probably no impact: 1/5 (20%) • Probably increase: 1/5 (20%) • Increased: 3/5 (60%)	Increased	“As T2DM disproportionately affects populations that are socioeconomically or otherwise disenfranchised/disadvantaged, we believe earlier screening can improve equity in T2DM care”
**Acceptability**	• Probably Yes: 3/5 (60%) • Yes: 2/5 (40%)	Probably Yes	“There may be resistance at the level of the patient/general population to engage in earlier T2DM screening, particularly considering certain stigmas associated with the diagnosis of T2DM.”
**Feasibility**	• Probably Yes: 1/5 (20%) • Yes: 4/5 (80%)	Yes	“Although limited healthcare access is a major concern for implementation of screening recommendations, T2DM screening is a basic test that is likely available at most testing facilities around the country, including in rural locations.”

An essential and basic principle of the development of any evidence-based CPGs is a comprehensive, robust, and transparent methodology, as this affects the accuracy, credibility, trustworthiness, and uptake of recommendations (25, 26). In addition to the general challenges faced by LMICs in developing and disseminating CPGs, the Pakistani health system has struggled in achieving mainstream uptake of CPGs by physicians in the country. Initiatives to create CPGs for the management of T2DM have been undertaken in Pakistan, such as the Pakistan Endocrine Society (PES) guidelines (2020) (27) and the PROMPT (Pakistan’s Recommendations for Optimal Management of diabetes from Primary to Tertiary care level) CPGs (created in 1999; revised in 2017) (28). However, these have not achieved nationwide penetrance into routine clinical practice (28). Though both refer to numerous international CPGs, they do not explicitly explain the methodologies used to assimilate and synthesize recommendations, with the PES guidelines simply stating that their CPGs were “*based on available local, regional and international scientific evidence including special considerations to affordability and availability of medicines in Pakistan and consensus statements by Guidelines committee of PES*”. This ambiguity in the processes followed in the development of the CPGs may underlie their underwhelming uptake locally. Thus, the use of adolopment to generate CPGs for the management of T2DM in Pakistan has the potential to address local challenges on multiple fronts. In addition to being context sensitive and resource efficient, adolopment incorporates granular detail and documentation that is informative and can be understood, appraised, and ultimately accepted by healthcare providers across the country.

The management of T2DM has increasingly become a responsibility of primary care/general practitioners (GPs) as disease burden in Pakistan increases, and the evidence suggests that patient outcomes may remain at par with specialist care if there is an emphasis on regular follow-ups and the use of quality management guidelines (29). Consequently, there is immense need for local T2DM CPGs to be developed for use by GPs, by following a transparent, standardized process that makes use of existing available best-evidence CPGs with appropriate context-specific modifications. Such CPGs would bring the healthcare system of Pakistan a step closer to achieving optimal health outcomes in T2DM and would have greater credibility by virtue of their transparent development processes. Thus, we aimed to employ the GRADE-ADOLOPMENT process to develop local evidence-based CPGs for the management of adult DM by GPs in Pakistan. Furthermore, our goal was to transparently present our methodology for benefits that are twofold. Firstly, researchers in high-income settings can observe how their work is adapted for use in regions like Pakistan. Secondly, guideline creators in LMICs, particularly in South Asia, can build upon and adapt our work to provide CPGs for use in their own local contexts.

## Methods

### Setting

This process was conducted at the CITRIC (Clinical and Translational Research Incubator) Center for Clinical Best Practices (CCBP) at the Aga Khan University (AKU) Hospital, Pakistan. The AKU is a private sector, not‐for‐profit hospital in Pakistan, and is also the country’s leading healthcare and biomedical research facility (30).

The CITRIC CCBP at AKU is tasked with the adaptation and development of evidence-based CPG and care pathways to standardize and improve healthcare in Pakistan. The GRADE-ADOLOPMENT processes described in this study have been implemented by the CCBP, in collaboration with the expertise of the Section of Endocrinology at AKU and the GRADE-USA working group, in the development of adult T2DM management CPGs for use by general practitioners (GPs)/primary care physicians in Pakistan. The decision to create T2DM CPGs for GPs rather than specialist endocrinologists is due to the growing prevalence of T2DM and the lack of specialists in Pakistan (31).

### Study team

The study team is comprised of the CCBP research staff (who are trained in GRADE methodology and in the development of management CPGs) as well as endocrinology faculty led by Endocrinology Section Head of AKU.

### Source guideline selection

The source guideline is the single, original, “parent” CPG that undergoes the GRADE-ADOLOPMENT process in the development of a local CPG. The *Standards of Medical Care in Diabetes – 2021 (Abridged for Primary Care Providers)* (5) was selected by the Section of Endocrinology as the source CPG, due to its comprehensive set of recommendations, integrated approach to management, and high-quality synthesis of available evidence.

The original recommendations within this CPG have been formulated by the American Diabetic Association (ADA) in association with the Grading of Recommendations Assessment, Development and Evaluation (short GRADE) working group which uses an established and transparent approach to grading quality (or certainty) of evidence and strength of recommendations.

### Guideline review

**Figure 1:**
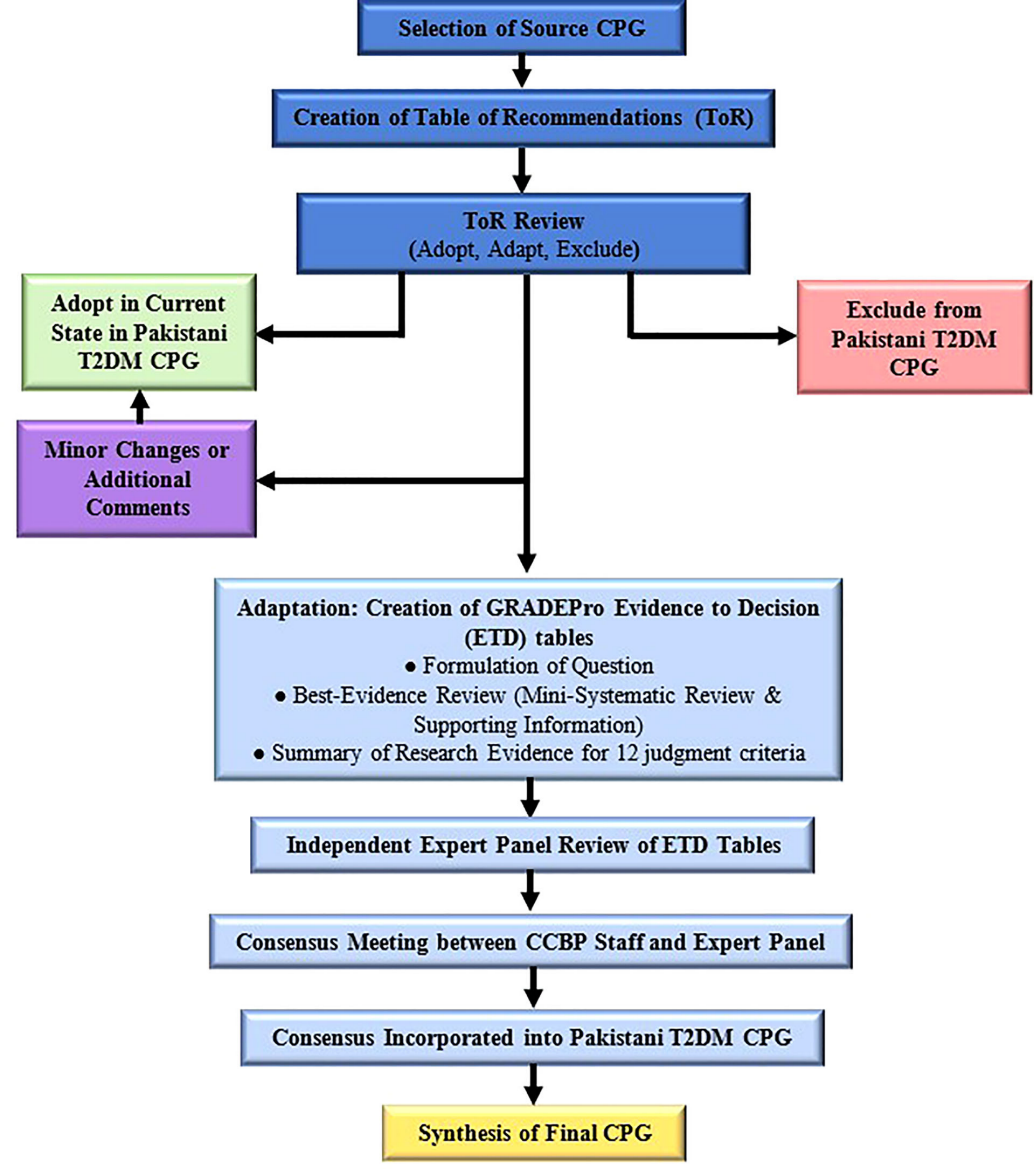
GRADE-ADOLOPMENT process for T2DM CPG for Pakistan.

**Figure 1** delineates the adolopment process used in our study. First, a Table of Recommendations (ToR) was created by extracting and compiling all recommendations mentioned in the source CPG. Two senior attending endocrinologists reviewed the ToR independently and marked each recommendation as either “*Adopt*,” “*Adapt”* or “*Exclude*.” Discrepancies were settled in consensus with the Section Head of Endocrinology. Recommendations marked “*Adopt*” were incorporated as is or with minor changes into the local CPG, while those marked “*Exclude*” were omitted from the local CPG. Exclusion was on the basis of the recommendation pertaining to pediatric or inpatient management, or if the recommendation was deemed irrelevant to the local Pakistani context. Other reasons for exclusion were required to be explained by the reviewers. It is important to note that recommendations pertaining to adult type 1 and gestational DM were not excluded.

Recommendations marked “*Adapt*” were deemed to warrant additional review and revision *via* the GRADE-ADOLOPMENT process (detailed below) before incorporation into the local CPGs. Our adolopment process (**Figure 1**) had two important differentiations to the one described originally (11). Firstly, we did not create any recommendations *de novo*, which was due to a lack of perceived need for additional recommendations, as well as the lack of resources and sufficient local research available. Secondly, recommendations that were deemed to require only minor and straightforward changes prior to adoption were not subjected to the complete adaptation process consisting of ETD tables and expert panel review.

### GRADEPro evidence to decision framework

GRADEPro is a web application used to create, manage, and share summaries of research evidence (32). The CCBP staff involved in this study underwent a training module to master use of GRADEPro for the GRADE-ADOLOPMENT process. The software was used to develop Evidence to Decision (ETD) tables to reach a consensus on each of the recommendations marked “*Adapt*.”

ETD tables are frameworks that enable members of an expert panel to make healthcare recommendations or decisions based on summarized, balanced, evidence. Development of ETD tables begins with formulation of a question structured as follows: “Should the *Intervention/Suggested Change* be favored over the *Comparison/Current Standard of Practice?*” The pros and cons of the suggested change are judged by an expert panel across 12 criteria, that are shown in **Supplementary Table 1**.

Each criterion was supported with evidence gathered through a best evidence review process (**Supplementary Material**), to provide local context for the pros and cons of the recommendation. The CCBP team summarized the newly gathered evidence for each criterion in the “*Research Evidence*” and “*Additional Considerations*” columns.

### Expert panel review

An expert panel of five endocrinology faculty were invited by the Endocrinology Section Head to review the completed ETD table for each recommendation and provide their judgement for each criterion. These experts are well versed in and have practiced previously using the American Diabetic Association guidelines (5), and the American Association of Clinical Endocrinologists and American College of Endocrinologists guideline (6).This judgment was in the form of a single selection from multiple response options. If, for any criteria, an expert required additional evidence, they informed the CCBP team. An effort was made to source the requisite information, which, if found, was shared with all the panel members. Experts’ judgements were sought in an anonymous and confidential manner, with the GRADEPro software allowing reviewers to select options and provide feedback without their identity known to fellow experts or the CCBP team. A dummy version of a GRADEPro ETD is shown as **Supplementary Table 2**.

### Final recommendation revisions and synthesis

Once all the members of the expert panel had provided their responses to the ETD, the CCBP staff synthesized their responses to produce a summary of judgments. The CCBP staff conducted a meeting with the expert panel to review the summary of judgments and reach a final unanimous consensus on the need for and nature of any revisions to the recommendations in question. The strength of each recommendation was also decided. Finally, the consensus was presented to the Section Head of Endocrinology for review, after which the recommendation was incorporated into the Pakistani CPG with a summary of the consensus decision.

### Final debriefing to identify challenges and explore solutions

Two focus group discussions (FGDs) were conducted to identify challenges faced throughout the entire GRADE-ADOLOPMENT process and to explore corresponding solutions. These FGDs were led by a member of the CCBP team and included the CCBP staff and the Section Head of Endocrinology. Participants were given the opportunity to first brainstorm challenges and solutions independently, and these were then discussed within the FGD. Each challenge was decided as per consensus opinion to be either a major or minor challenge. The CCBP team then categorized the final list of specific challenges within broad themes, and their corresponding solutions were presented alongside them.

### Timeline of creation of T2DM CPG for Pakistan

The methodology described in this study was executed according to the following timeline, spanning November 2021 – April 2022:

- Source Guideline Selection: November 2021 (3 weeks)
- Creation of Table of Recommendations: November 2021 (3 weeks)
- Review of Table of Recommendations: December 2021-January 2022
- Identification of recommendation for adaptation: January 2022 (2 weeks)
- Creation of the ETD table: February-March 2022
- Expert panel identification and independent review of the ETD table: March 2022 (1 month)
- Combined consensus meeting between CCBP staff and Expert panel: April 2022 (2 hours)
- Consensus incorporated into the Pakistani T2DM CPG: April 2022 (1 day)
- Creation of final Pakistani T2DM CPG: April 2022 (2 weeks)

## Results

### Initial review of source guideline

**Figure 2:**
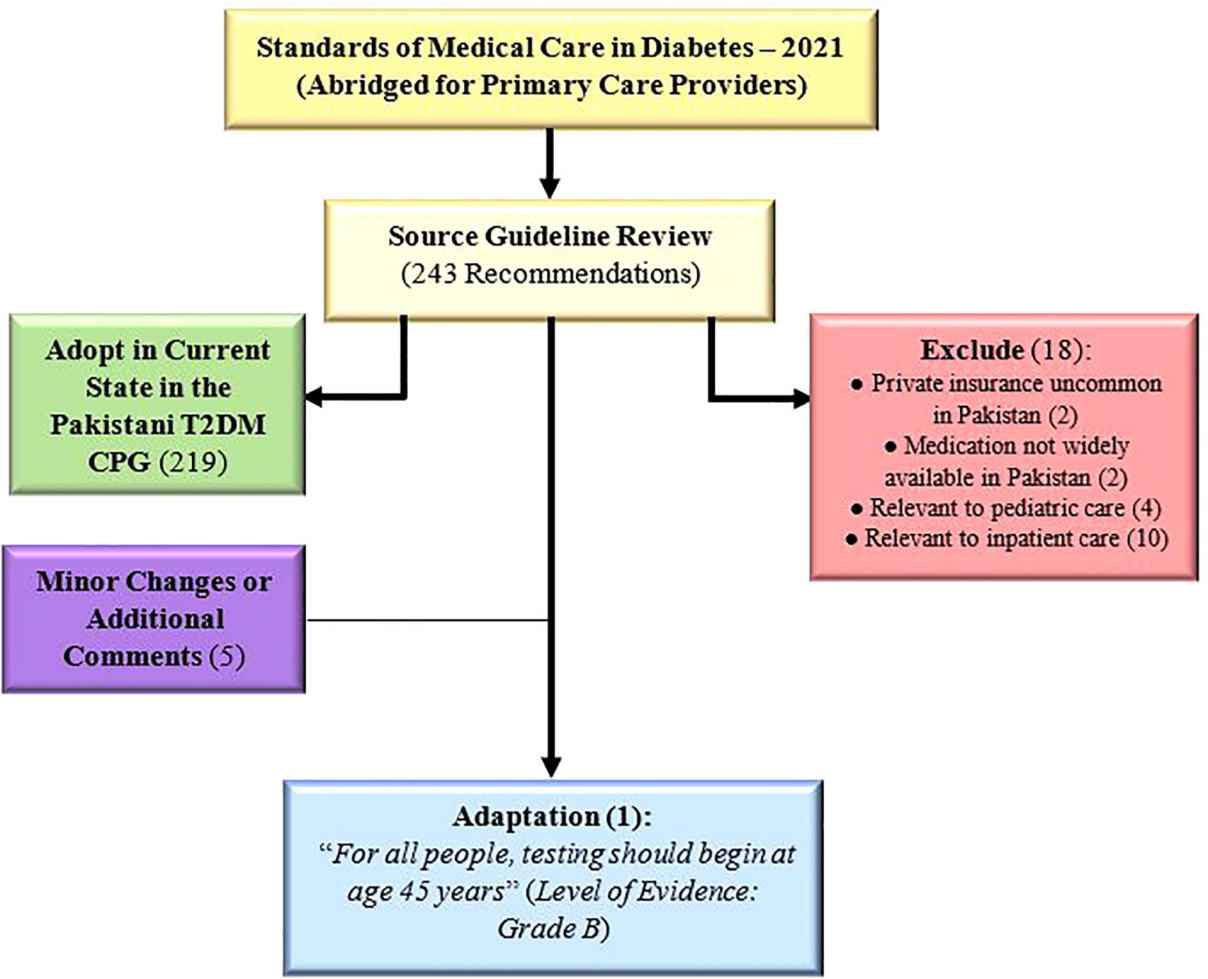
Outcomes for GRADE-ADOLOPMENT process.

The source guideline (5) included a total of 243 recommendations, out of which 219 were adopted as is, 5 were adopted with minor changes/additional comments (**Supplementary Table 3**), and 18 were excluded. Only one recommendation (2.9 in source guideline) was deemed to require adaptation: “*For all people, testing should begin at age 45 years*” (**Figure 2**). Amongst the 18 excluded recommendations, most were excluded because they were applicable to inpatient (n=10) or pediatric care (n=4), with the remaining excluded due to the lack of private insurance (n=2) or medication availability (n=2) in Pakistan (**Supplementary Table 4**).

### Evidence-to-decision table

The full-length ETD table, along with all the research evidence and additional considerations provided to the expert panel, is presented in **Supplementary Table 5**. The summary of independent expert judgments and final group consensuses for each of criterion is shown in **Table 1**. The ETD elicited the experts’ opinion on whether the 12 criteria favored the intervention (screening before the age of 45 years) or the comparison/control (screening after the age of 45 years).

Amongst the 12 criteria, “*Problem*” had a 100% agreement (“*yes*”), while 80% agreement was achieved for “*Desirable Effects*” (“*large*”), “*Undesirable Effects*” (“*small*”), “*Feasibility*” (“*yes*”). All other criteria had 60% agreement, barring “*Cost-Effectiveness*” where 40% felt it “*favored the comparison*”, 40% felt it “*favored the intervention*”, and 20% felt that it “*probably favored the intervention*”. However, the consensus on “*Cost-Effectiveness*” after the final meeting of the CCBP with the expert panel was that it “*favored the intervention*”.

### Challenges and solutions

The challenges faced were broadly categorized into four main themes: resources, stakeholder support and involvement, resistance to change, and methodological limitations (**Table 2**).

**Table 2:** Challenges faced and proposed solutions.

Category of Challenge	Specific Challenge	Proposed Solution
**Resources**	• Inadequate original data from Pakistan: There are not enough original articles published based on the Pakistani population **	• Make use of regional literature • Judicious use of grey-literature
	• Structuring the GRADE-ADOLOPMENT process to the resource-constrained context of Pakistan (revise through experience, highlight resource gap): It was challenging to reform the GRADE-ADOLOPMENT process for use due to restricted resources **	**•** Conduct a thorough, realistic resource assessment, and highlight resource gaps • Revise process accordingly through experience
	• Inadequate expertise and experience with guideline development: It was challenging to find guideline creating experts *	**•** Collaborate with personnel with requisite experience and expertise **•** Conduct comprehensive, standardized training modules for all personnel involved in the adolopment
	• Inadequate manpower/size of workforce: The workforce was limited due to resource constraints *	**•** Involve students and trainees on a volunteer basis
	**•** Suboptimal funding: The project received suboptimal funding to create guidelines *	**•** Lobby for additional institutional and external funding
**Stakeholder Support and Involvement**	• Coordination between different stakeholders: It was challenging to coordinate with multiple stakeholders at the same time **	**•** Fixed, scheduled meetings with regular follow-ups with all stakeholders (Awareness presentations/meeting directly)
	• Suboptimal departmental support: It was challenging to incentivize expert involvement **	• Involve all stakeholders from the start • Emphasize and reiterate mutual interests • Design specific curricula for all stakeholders involved • Tailor and deliver presentations to all stakeholders involved
	• Suboptimal multidisciplinary departmental collaboration: It was challenging to incentivize experts for a multidisciplinary collaboration *	
	• Suboptimal provincial/federal government involvement: There was suboptimal support from the government *	
	• Suboptimal involvement of external societies or organizations: There was suboptimal support from external societies and organizations *	
	• Absence of patients’ perspective: Patients were not involved in the guideline creation process *	
	• Absence of general practitioners’ perspective: General practitioners were not involved in the guideline creation process *	
	• Absence of the allied health perspective: Allied health experts were not involved in the guideline creation process *	
	**•** Suboptimal institutional support: The project received suboptimal support *	
	• Conflicts of interest between different stakeholders (e.g., experts’ pharmaceutical ties, business potential of earlier laboratory testing and treatment at AKU): There was inevitable conflict of interest from different stakeholders *	• Involve stakeholders from diverse backgrounds • Mandate disclosure of all conflicts and preclude those with conflicts of interests from participating • Use generic drug names wherever possible
**Resistance to Change**	• Experts’ doubts regarding need for local CPGs: Some experts questioned the need of a new guideline for Pakistan**	• Initial presentation to emphasize need for local CPGs, robustness of the GRADE-ADOLOPMENT process, and the importance of strict adherence to rigorous GRADE-ADOLOPMENT processes in order to produce credible guidelines
	• Experts’ doubts regarding credibility of GRADE-ADOLOPMENT process: Some experts questioned the reliability of the process **	
	• Experts’ doubts regarding feasibility of GRADE-ADOLOPMENT process: Some experts questioned the practicality of the process **	
	• Rigorousness of the GRADE-ADOLOPMENT process may discourage the process of adaptation (rigor ensures robustness): Some experts may elude from the rigorous process of adaptation **	
	• Intellectual laziness on the part of experts: Some experts may not put in their maximum efforts **	• Provide protected time for experts to dedicate for all GRADE-ADOLOPMENT activities they are involved in
	**•** Experts’ exercising caution/opting for middle-ground with regards to decision-making in ETD table: Some experts may be extra cautious and hesitant to make drastic changes in existing recommendations **	• Emphasize the anonymity of the ETD process • Emphasize importance of incorporating varying schools of thought in the adaptation process
	• Institutional hierarchy in expert panel may have dissuaded explicit freedom of expression during consensus meeting: Some experts may be discouraged from stating their concerns freely due to the institutional hierarchy **	• Involve experts with similar proficiency and experience from different institutions • Involve a moderator in consensus meeting
	• Experts’ concerns regarding duplication of existing local guidelines: Some experts were concerned that the end result would be a duplicate of the source guideline *	• Initial presentation to highlight deficiencies of existing CPGs and need for comprehensive CPGs produced using transparent methodology
	• Experts’ doubts regarding nationwide implementation of local guidelines: Some experts questioned how well the local CPGs will be implemented *	• Involve decision-makers from other institutions across the country and ensure buy-in to the newly adoloped CPGs
**Methodological Limitations**	• Individual-level biases from experts: Some inevitable bias may arise at an individual level **	• Increase the number and diversity of experts • Gauge acceptability and accuracy of any revisions made by getting feedback from experts from external institutes
	• Group-level biases from experts: Some inevitable bias may arise at the group level **	
	• Suboptimal generalizability of consensus opinion based on 5 experts: The opinion of 5 experts would be hard to generalize widely **	
	• Expert opinion is no substitute for the lack of scientific evidence: Scientific evidence, or rather lack thereof, cannot be replaced by expert consensus *	• Supplement the expert opinion with as much auxiliary evidence as possible • Plan future studies to answer specific questions that arise during the GRADE-ADOLOPMENT process

* Minor challenge; ** Major Challenge.

## Discussion

In this paper we describe the GRADE-ADOLOPMENT process employed for the creation of evidence-based adult DM CPGs for Pakistan. We aimed to produce a CPG, using a rigorous and transparent methodology, suited to implementation in the local healthcare context of Pakistan. With the *Standards of Medical Care in Diabetes – 2021 (Abridged for Primary Care Providers)* (5) used as the source guideline, the adolopment process resulted in 1 adapted recommendation and 18 excluded recommendations. Minor changes or associated comments were attached to five of the remaining recommendations which were otherwise adopted directly.

The adoloped adult DM CPG for Pakistan recommends initiation of screening for DM/prediabetes in all Pakistanis after the age of 30 years, as opposed to the original recommendation of 45 years of age in the source CPG. Interestingly, the subsequent edition of our source guideline, the *Standards of Medical Care in Diabetes – 2022 Abridged for Primary Care Providers*, also updated its recommendation for screening, lowering the age to 35 years (33). This change was based off the US Preventive Services Task Force (USPSTF) statement in August 2021 regarding the need for and benefits of earlier screening (34). Our newly adapted recommendation also adds to current PES and PROMPT CPGs, which do not provide any explicit recommendations for the age after which individuals should be screened (27, 28). This is a significant omission for several reasons. The prevalence of T2DM in Pakistan is an overwhelming 26.7%, as reported by the IDF (3). Of these patients with T2DM, over 25% are aged <40 years and 50% are between 40 and 59 years of age (35). Thus, a large proportion of patients in Pakistan developed T2DM, or were at risk of developing T2DM, before the age of 45 years. Therefore, the age at which screening ought to be initiated must be stated and correspondingly lowered to enable the early detection and management of T2DM. In India, a LMIC with similar T2DM prevalence, the current guidelines in use by the National Program for Prevention and Control of Cancer, Diabetes, Cardiovascular Disease and Stroke (NPCDCS) states that the screening process for T2DM should be initiated at the age of 30 years (36).

It is well established that individuals originating from South Asian countries like India, Bangladesh, Sri Lanka, and Pakistan are at a particularly high risk of suffering from T2DM and its multitude of complications. This increased risk of disease and associated morbidity is attributed to factors such as poor maternal nutrition and impaired intrauterine growth, high rates of childhood obesity, and ever-increasing proportions of fats and sugars in typical diets in these regions (37). Early detection and appropriate management of T2DM is thus crucial for preventing avoidable morbidity and mortality, particularly its debilitating late-stage multi-organ complications in the Pakistani and other South Asian populations. Without region-appropriate age cut-offs for screening of T2DM based on epidemiological evidence, countermeasures largely take the form of tertiary prevention, whereby patients with T2DM present due to the onset of systemic complications (38). These include diabetic eye disease, neuropathy, peripheral arterial disease (with manifestations including diabetic foot ulcers and delayed wound healing) (39). By this point the disease may have taken a severe and frequently irreversible toll on their health, functionality, and quality of life. By initiating screening after the younger age of 30 years in Pakistan, patients and their physicians may act earlier and more effectively to slow disease progression and avoid serious complications (40). Apart from benefiting from earlier pharmacologic intervention, younger patients are also more likely in general to be able to implement lifestyle modifications and are more proactive regarding their healthcare (40–42). Earlier screening highlights the opportunity to intervene early on and reduce complications. It also represents a greater opportunity to promote physical activity to patients in Pakistan, emphasizing the essential role of aerobic exercise and resistance training in enhancing insulin sensitivity to maintain and restore glycemic control (1).

Of particular concern are the economic costs borne by individuals and the community because of the effects of T2DM associated morbidity on their careers and productivity (43). T2DM debilitates individuals and induces complications that hinder their ability to work jobs and earn a living, with a particularly aggressive disease phenotype in cases with earlier onset (44). Furthermore, younger patients with T2DM have severe stress and impaired emotional well-being, with greater rates of depression and fear (45). The possible consequences of such psychological burdens on patients’ social lives, including activities like parenting, as well as mental health and quality of life (46), are concerning. Thus, with its alarmingly high prevalence in Pakistanis of a working age, T2DM represents a burden on all spheres of these patients’ lives and likely causes economic costs to individual patients, their families and communities, and the country. The monetary losses borne by patients quickly add up when considering the loss of time, health, and productivity, as well as the expenditure in procuring drugs, devices for CGM (continuous glucose monitoring) such as glucometers (47), or simply seeing a physician. In this vein, implementation of screening after the age of 30 has the potential to be cost saving to patients (48, 49), a crucial benefit in the LMIC setting of Pakistan, where most healthcare expenditure is out-of-pocket (50–52). This lack of health insurance (or other risk pooling systems of healthcare financing) is also thus a reason for excluding one of the recommendations from the source guideline (**Figure 2**). Physicians must consider the high cost of care when planning management with patients in LMICs, noting particularly the fact that urgent and expensive interventions for late-stage complications confer a greater financial risk (53, 54). Furthermore, the adopted recommendations involving CGM were coupled with the acknowledgement that obtaining glucometers or similar technology may not be economically feasible for patients in Pakistan. Affordability of care is indeed a crucial point of consideration in T2DM care in an LMIC setting, as burdensome medical expenses can negatively impact patients’ lives or interfere with their adherence to therapy.

A crucial aspect of developing evidence-based CPGs is to use a rigorous and transparent methodology. A systematic and transparent approach is more likely to produce effective and feasible guidelines with greater credibility and adoption by local physicians (25, 26). The absence of documented and transparent methods, beyond cursory details, may explain the low utilization of existing guidelines for the management of DM in Pakistan (28). It may also explain why neither of the currently available sets of CPGs address the matter of screening, despite the evidence pointing to it requiring alteration to a younger recommended age in Pakistan. The GRADE-ADOLOPMENT method overcomes this flaw by grounding its output in a high-quality source CPG created by highly qualified experts who spared no resource in systematically analyzing all the available literature to form recommendations.

The GRADE-ADOLOPMENT method has the added advantage of identifying key avenues for future research. Focusing areas of study can generate evidence while still working within resource constraints, without incurring costs or dedicating resources or personnel to generating evidence that is unlikely to influence policy or clinical decision making. The level of evidence supporting our adapted guideline is a GRADE B as per the GRADEPro methods used (55). Future research should explore the effectiveness of screening earlier to provide higher quality of evidence to support or refute the new recommendation. Additionally, the research and development of new drugs and tools for DM must be more mindful of the target population of diabetics, which primarily resides in LMICs as stated earlier. Though of great value in wealthier countries, tools such as CGM devices can only be as impactful as they are accessible. Their relative costliness highlights the need for affordable technologies and treatment modalities that can achieve widespread adoption in LMICs to reach most diabetic patients, who live in conditions of financial constraints. To sum up, a systematic and disclosed methodology produces credible guidelines more likely to achieve high penetrance locally, while also guiding future research and facilitating a continuous process of improvement over time as the availability of evidence improves and the disease profile of T2DM in Pakistan evolves.

There are limitations to our adolopment process and the newly adoloped Pakistani T2DM CPG that we wish to acknowledge. Fundamental to the GRADE-ADOLOPMENT process, the revision made to age of screening initiation was based on expert consensus informed by suboptimal level of evidence. Moreover, we did not include other important stakeholders, such as patients, allied health professionals, general practitioners, nurses, experts external to AKU, other healthcare centers, external organizations or societies concerned with DM management, and provincial and federal governments. The decision to limit widespread stakeholder involvement was to minimize undue delays stemming from factors including logistic difficulties, conflicts of interest, lack of mutual availability, political influences, and lack of direct incentives. These factors represent real-world barriers to the implementation of the ideal GRADE-ADOLOPMENT process, especially in LMICs like Pakistan. Moreover, prior experience in developing such CPGs enabled the CCBP team to remain mindful of the needs and values of these groups to a large extent. Future efforts may include the development of ‘*post-hoc*’ additions from these stakeholders to be incorporated into future addendums or iterations of the DM guidelines.

Moreover, while the benefits of early screening for T2DM in Pakistan are numerous, the feasibility of such practice remains to be seen with particular concern for rural implementation. Rural locations in Pakistan often lack the infrastructure and healthcare facilities needed to provide screening services. Similar logistical hurdles were the basis for excluding several of the guidelines from the parent CPGs. For instance, the recommendation to prescribe glucagon for patients at risk of episodes of hypoglycemia, a life-saving medical standard in developed countries, was excluded due to the lack of availability of glucagon in Pakistan. Financial hurdles hinder the feasibility of widespread use of CGM devices in a similar fashion. Thus, implementing screening for patients above the age of 30 years can be a challenge for Pakistan’s health sector. Low accessibility, whether physical or in a financial sense, also adds to difficulties experienced by patients. Given the LMIC context of Pakistan, and a general lack of awareness of the morbidity and mortality associated with T2DM, convincing individuals to participate actively in screening may prove challenging, even if the exercise is cost-saving to patients in the long term.

## Conclusion

This paper reports the adoloped T2DM CPG for adults in Pakistan, which was developed using a transparent and rigorous methodology which we have documented. The CPG recommends screening for T2DM above the age of 30 years, which is a modification from its source guideline by the American Diabetes Association. This lower cut-off for age at which screening should be initiated, is justified primarily by the high prevalence of T2DM amongst younger individuals in South Asia, particularly Pakistan. 18 recommendations in the source document were excluded, either because they pertained to pediatric or inpatient recommendations, or due to unavailability of certain drugs in Pakistan, or the lack of private insurance. General Practitioners in Pakistan can implement these guidelines in their practices knowing they are applicable to the local setting based on sound evidence. The nationwide implementation of the adoloped guidelines will make T2DM care more context-specific and equitable. Furthermore, groups in similar settings may review our methodology and process to adopt a similar approach to developing CPGs suitable for their local health systems. Future research should explore the effectiveness of earlier T2DM screening in a Pakistani population.

## Data availability statement

The original contributions presented in the study are included in the article/**Supplementary Material**. Further inquiries can be directed to the corresponding author.

## Author contributions

MQM, NAR, AS, NJ, AK, NR, SF, MAM, SA, AH, and SN were involved in the conceptualization and creation of the adoloped evidence-based clinical practice guideline. RM, OM, AP and SN were involved in the conceptualization of the manuscript and writing of its first draft. All authors contributed to the article and approved the submitted version.

## References

[1] Zheng Y Ley SH Hu FB. Global aetiology and epidemiology of type 2 diabetes mellitus and its complications. Nat Rev Endocrinol (2018) 14(2):88–98. doi: 10.1038/nrendo.2017.151 29219149

[2] Flood D Seiglie JA Dunn M Tschida S Theilmann M Marcus ME. The state of diabetes treatment coverage in 55 low-income and middle-income countries: A cross-sectional study of nationally representative, individual-level data in 680 102 adults. Lancet Health Longevity. (2021) 2(6):e340–e51. doi: 10.1016/S2666-7568(21)00089-1 PMC8865379 35211689

[3] Federation ID. International Diabetes Federation. IDF Diabetes Atlas, 10th edition. Brussels, Belgium: IDF diabetes atlas. (2021). Available at: https://www.diabetesatlas.org.

[4] Hussain A Ali I. Diabetes mellitus in Pakistan: A major public health concern. Peshawar, Pakistan: Archives of Pharmacy Practice, Vol. 7. (2016). pp. 30–3.

[5] Association AD. Standards of medical care in diabetes–2021 abridged for primary care providers. Clin diabetes: Publ Am Diabetes Assoc (2021) 39(1):14. doi: 10.2337/cd21-as01 PMC7839613 33551551

[6] Handelsman Y Bloomgarden ZT Grunberger G Umpierrez G Zimmerman RS Bailey TS. American Association of clinical endocrinologists and American college of endocrinology–clinical practice guidelines for developing a diabetes mellitus comprehensive care plan–2015—executive summary. Endocrine Pract (2015) 21(4):413–37. doi: 10.4158/EP15672.GL 27408942

[7] Guideline N. Type 2 diabetes in adults: Management (update). United Kingdom: National Institute for Health and Care Excellence (2022). 32023018

[8] Anwer MA Al-Fahed OB Arif SI Amer YS Titi MA Al-Rukban MO. Quality assessment of recent evidence-based clinical practice guidelines for management of type 2 diabetes mellitus in adults using the AGREE II instrument. J Eval Clin Pract (2018) 24(1):166–72. doi: 10.1111/jep.12785 28948654

[9] Diabetes Canada. Diabetes Canada 2018 clinical practice guidelines for the prevention and management of diabetes in Canada: Diabetes Canada. (2018).

[10] Zhao X Guo L Lin Y Wang H Gu C Zhao L. The top 100 most cited scientific reports focused on diabetes research. Acta Diabetol (2015) 53:13–26. doi: 10.1007/s00592-015-0813-1 26596851

[11] Schünemann HJ Wiercioch W Brozek J Etxeandia-Ikobaltzeta I Mustafa RA Manja V. GRADE evidence to decision (EtD) frameworks for adoption, adaptation, and *de novo* development of trustworthy recommendations: GRADE-ADOLOPMENT. J Clin Epidemiol (2017) 81:101–10. doi: 10.1016/j.jclinepi.2016.09.009 27713072

[12] Ali MK Seiglie JA Narayan KMV. Progress in diabetes prevention or epidemiology-or both, or neither? Lancet Diabetes Endocrinol (2021) 9(4):190–1. doi: 10.1016/S2213-8587(20)30433-2 PMC8010713 33636103

[13] Hussain M Baqir S Naqvi S Khan MA Rizvi M Alam S. Direct cost of treatment of diabetes mellitus type 2 in Pakistan. Int J Pharm Pharm Sci (2014) 6:261–4.

[14] Zia A Bhatti A Jalil F Wang X John P Kiani A. Prevalence of type 2 diabetes–associated complications in Pakistan. Int J Diabetes Develop Countries (2015) 36:179–88.

[15] Liaqat A Arshad S Gul S Sultana U Liaqat F Liaqat A. Comparison of social determinants and evaluation of disease management of diabetic patients attending rahman medical institute and nahaki emergency satellite hospital, peshawar. Pakistan J Public Health (2021) 11(1):30–4. doi: 10.32413/pjph.v11i1.635

[16] Iqbal Q Ul Haq N Bashir S Bashaar M. Profile and predictors of health related quality of life among type II diabetes mellitus patients in quetta city, Pakistan. Health Qual Life outcomes. (2017) 15(1):142. doi: 10.1186/s12955-017-0717-6 28709437 PMC5512812

[17] Gillani AH Amirul Islam FM Hayat K Atif N Yang C Chang J. Knowledge, attitudes and practices regarding diabetes in the general population: A cross-sectional study from Pakistan. Int J Environ Res Public Health (2018) 15(9):1906. doi: 10.3390/ijerph15091906 30200534 PMC6164838

[18] Kurji Z Premani ZS Mithani Y. Analysis of the health care system of Pakistan: Lessons learnt and way forward. J Ayub Med College Abbottabad JAMC. (2016) 28(3):601–4. 28712245

[19] Bahadar H Mostafalou S Abdollahi M. Growing burden of diabetes in Pakistan and the possible role of arsenic and pesticides. J Diabetes Metab Disord (2014) 13(1):117. doi: 10.1186/s40200-014-0117-y 25530951 PMC4271443

[20] Haque Z Javed A Victor S Mehmood A. Attitudes and behaviour of adult Pakistani diabetic population towards their disease. J Dow Univ Health Sci (JDUHS). (2014) 8(3):89–93.

[21] Tugwell P Knottnerus JA. Adolopment - a new term added to the clinical epidemiology lexicon. J Clin Epidemiol. (2017) 81:1–2. doi: 10.1016/j.jclinepi.2017.01.002 28215275

[22] Andrews JC Schünemann HJ Oxman AD Pottie K Meerpohl JJ Coello PA. GRADE guidelines: 15. going from evidence to recommendation–determinants of a recommendation's direction and strength. J Clin Epidemiol (2013) 66(7):726–35. doi: 10.1016/j.jclinepi.2013.02.003 23570745

[23] Alonso-Coello P Oxman AD Moberg J Brignardello-Petersen R Akl EA Davoli M. GRADE evidence to decision (EtD) frameworks: A systematic and transparent approach to making well informed healthcare choices. 2: Clinical practice guidelines. BMJ (2016) 353:i2089. doi: 10.1136/bmj.i2016 27365494

[24] Alonso-Coello P Schünemann HJ Moberg J Brignardello-Petersen R Akl EA Davoli M. GRADE evidence to decision (EtD) frameworks: A systematic and transparent approach to making well informed healthcare choices. 1: Introduction. Bmj (2016) 353:i2016. doi: 10.1136/bmj.i2016 27353417

[25] Steinberg E Greenfield S Wolman DM Mancher M Graham R. Clinical practice guidelines we can trust. Washington DC, United States: National Academies Press (2011). 24983061

[26] Qaseem A Forland F Macbeth F Ollenschläger G Phillips S van der Wees P. Guidelines international network: toward international standards for clinical practice guidelines. Ann Internal Med (2012) 156(7):525–31. doi: 10.7326/0003-4819-156-7-201204030-00009 22473437

[27] Khan KA. Pakistan Endocrine society (PES)-2020 guidelines for management of type IIdiabetes mellitus and cardiometabolic syndrome. JPMA (2020) 70(11):S–1–8.

[28] Shera AS Basit A Team P. Pakistan’s recommendations for optimal management of diabetes from primary to tertiary care level (PROMPT). Pakistan J Med Sci (2017) 33(5):1279. doi: 10.12669/pjms.335.13665 PMC5673748 29142579

[29] Renders CM Valk GD Griffin SJ Wagner EH Eijk van JT Assendelft WJJ. Interventions to improve the management of diabetes in primary care, outpatient, and community settings: A systematic review. Diabetes Care (2001) 24(10):1821–33. doi: 10.2337/diacare.24.10.1821 11574449

[30] Haq IU Rehman ZU. Medical research in pakistan; a bibliometric evaluation from 2001 to 2020. Library Philosophy Pract (2021), 1–13.

[31] Bhatti MW. ‘Country facing shortage of internal medicine specialists owing to non-practising female doctors'. News (2022).

[32] GRADEpro G. GRADEpro GDT: GRADEpro guideline development tool [Software]. McMaster University: Evidence Prime, Inc (2015).

[33] Association AD. Standards of medical care in diabetes–2022 abridged for primary care providers. Clin Diabetes. (2022) 40(1):10–38. doi: 10.2337/cd22-as01 35221470 PMC8865785

[34] Force UPST. Screening for prediabetes and type 2 diabetes: US preventive services task force recommendation statement. JAMA (2021) 326(8):736–43. doi: 10.1001/jama.2021.12531 34427594

[35] Federation ID Atlas I International Diabetes Federation. IDF diabetes atlas. 6th. Brussels, Belgium: International Diabetes Federation (2013).

[36] India DGoHSMoHFwGO. National programme for prevention and control of cancer, diabetes, cardiovascular diseases & stroke (NPCDCS). India: Directorate General of Health Services Ministry of Health & Family Welfare, Government Of India (2017).

[37] Praveen PA Kumar SR Tandon N. Type 2 diabetes in youth in south Asia. Curr Diabetes Rep (2015) 15(2):571. doi: 10.1007/s11892-014-0571-4 25620404

[38] Shrestha N Mishra SR Ghimire S Gyawali B Mehata S. Burden of diabetes and prediabetes in Nepal: A systematic review and meta-analysis. Diabetes Ther (2020) 11(9):1935–46. doi: 10.1007/s13300-020-00884-0 PMC7434818 32712902

[39] Qureshi SS Amer W Farooq M Butt N Shoaib Z Firdous S. Clinical presentations of type II diabetes. Pakistan J Med Health Sci (2017) 11:108–10.

[40] Hafeez A Rehman K Rehman A Ch AH. Diabetes mellitus: Morbidity and mortality in diabetics at independent university hospital, faisalabad, pakistan. Prof Med J (2018) 25(09):1406–12. doi: 10.29309/TPMJ/2018.25.09.143

[41] Buda ES Hanfore LK Fite RO Buda AS. Lifestyle modification practice and associated factors among diagnosed hypertensive patients in selected hospitals, south Ethiopia. Clin Hypertension. (2017) 23(1):26. doi: 10.1186/s40885-017-0081-1 PMC5713156 29214054

[42] DeVoe JE Wallace LS Fryer GE Jr. Patient age influences perceptions about health care communication. Family Med (2009) 41(2):126–33. PMC4918755 19184691

[43] Vijan S Hayward RA Langa KM. The impact of diabetes on workforce participation: Results from a national household sample. Health Serv Res (2004) 39(6p1):1653–70. doi: 10.1111/j.1475-6773.2004.00311.x PMC1361091 15533180

[44] Magliano DJ Sacre JW Harding JL Gregg EW Zimmet PZ Shaw JE. Young-onset type 2 diabetes mellitus–implications for morbidity and mortality. Nat Rev Endocrinol (2020) 16(6):321–31. doi: 10.1038/s41574-020-0334-z 32203408

[45] Wilmot E Idris I. Early onset type 2 diabetes: Risk factors, clinical impact and management. Ther Adv chronic Dis (2014) 5(6):234–44. doi: 10.1177/2040622314548679 PMC4205573 25364491

[46] Feng X Astell-Burt T. Impact of a type 2 diabetes diagnosis on mental health, quality of life, and social contacts: a longitudinal study. BMJ Open Diabetes Res Care (2017) 5(1):e000198. doi: 10.1136/bmjdrc-2016-000198 PMC5316913 28243446

[47] Nadeem S Siddiqi U Martins RS Badini K. Perceptions and understanding of diabetes mellitus technology in adults with type 1 or type 2 DM: A pilot survey from Pakistan. J Diabetes Sci Technol (2021) 15(5):1052–8. doi: 10.1177/19322968211011199 PMC8442186 33957791

[48] Echouffo-Tcheugui JB Ali MK Griffin SJ Narayan KMV. Screening for type 2 diabetes and dysglycemia. Epidemiol Rev (2011) 33(1):63–87. doi: 10.1093/epirev/mxq020 21624961

[49] Kahn R Alperin P Eddy D Borch-Johnsen K Buse J Feigelman J. Age at initiation and frequency of screening to detect type 2 diabetes: A cost-effectiveness analysis. Lancet (2010) 375(9723):1365–74. doi: 10.1016/S0140-6736(09)62162-0 20356621

[50] Statistics PBo. Government of Pakistan bureau of statistics Karachi. Pakistan: Pakistan Bureau of Statistics (2021).

[51] Cheema AR Zaidi S Najmi R Khan FA Kori SA Shah NA. Availability does not mean utilisation: analysis of a large micro health insurance programme in Pakistan. Global J Health Sci (2020) 12(10):1–4. doi: 10.5539/gjhs.v12n10p14

[52] Khalid F Raza W Hotchkiss DR Soelaeman RH. Health services utilization and out-of-pocket (OOP) expenditures in public and private facilities in Pakistan: an empirical analysis of the 2013–14 OOP health expenditure survey. BMC Health Serv Res (2021) 21(1):1–14. doi: 10.1186/s12913-021-06170-4 33632234 PMC7905921

[53] Ganasegeran K Hor CP Jamil MFA Loh HC Noor JM Hamid NA. A systematic review of the economic burden of type 2 diabetes in Malaysia. Int J Environ Res Public Health (2020) 17(16):5723. doi: 10.3390/ijerph17165723 32784771 PMC7460065

[54] Einarson TR Acs A Ludwig C Panton UH. Economic burden of cardiovascular disease in type 2 diabetes: A systematic review. Val Health (2018) 21(7):881–90. doi: 10.1016/j.jval.2017.12.019 30005761

[55] Joanna Briggs Institute Levels of Evidence and Grades of Recommendation Working Party. Supporting document for the Joanna Briggs institute levels of evidence and grades of recommendation. (2014).

