# Supplementary material for: Adolopment of adult diabetes mellitus management guidelines for a Pakistani context: Methodology and challenges
Source: Front Endocrinol (Lausanne). 2023 Jan 5;13:1081361. doi: 10.3389/fendo.2022.1081361 (PMC9849674; doi:10.3389/fendo.2022.1081361)
Supplement: Supplementary file 1 [file Table_1.docx]

**Supplementary Material**

1. *Best Evidence Review Process*
2. *Supplementary Table 1: Criteria evaluated in the Evidence to Decision (ETD) tables*
3. *Supplementary Table 2: Dummy Evidence to Decision (ETD) table*
4. *Supplementary Table 3: Recommendations indicated for Exclusion*
5. *Supplementary Table 4: Summary of Evidence to Decision Table*
6. *Supplementary Table 6: Table of Recommendations*

**Best-Evidence Review**

A best-evidence review was conducted to source research evidence that would help a recommendation be assessed across all 12 criteria. The best-evidence review was conducted separately for each of the 12 criteria and included a mini-systematic review and review of supporting evidence.

- *Mini-Systematic Review*: A mini-systematic review follows the same general protocol as a full systematic review, but applies arbitrary selection criteria (such as geographical region of publication) or limits the number of databases searched ^(1)^. In our mini-systematic review, PubMed and Google Scholar were queried using a search string designed using keywords from the recommendation in question. To maintain a local focus, only articles reporting data relevant to Pakistan were selected.

Two members of CCBP staff independently screened the titles and abstracts of articles sourced from PubMed and Google, with only those reporting relevant information specific to Pakistan undergoing a full-text review to finalize inclusion. As the source guideline itself was produced based on a systematic review process, careful full-text review of the bibliography within the source document was conducted. Two members of the CCBP staff extracted appropriate evidence from the final list of articles included.

- *Supporting Evidence*: Information pertaining to the cost of different investigations and treatments, as well as the availability of diagnostic and management facilities, was sourced from a selection of local hospitals, healthcare facilities, and pharmacies via telephonic query and their websites.

| **Supplementary Table 1: Criteria evaluated in the Evidence to Decision (ETD) tables** | | |
| --- | --- | --- |
| **Criteria** | **Description** | **Interpretation** |
| **Problem** | The magnitude of a problem, as measured by its prevalence and severity in a local context | The more serious or urgent a problem is, the more likely that the option that better addresses the problem receives a strong recommendation. |
| **Desirable Effects** | The magnitude of desirable effects is judged by considering the importance of the outcome and the size of the desirable effects (likelihood of experiencing a benefit or degree of benefits an individual experiences). | An option with greater desirable effects is more likely to gain a strong recommendation. |
| **Undesirable Effects** | The magnitude of undesirable effects is similarly judged by considering the importance of the outcome and the size of the undesirable effects. | An option with fewer undesirable effects is more likely to gain a strong recommendation. |
| **Certainty of Evidence** | Determined by likelihood that the research provides valid evidence regarding the effect of the option on all critical outcomes. | Evidence with higher certainty lends to a strong recommendation. |
| **Values** | Magnitude of value is judged by the variability or uncertainty of weightage placed upon the outcome by individuals. | Less variability/uncertainty of value leads to a strong recommendation. |
| **Balance of Effects** | The balance of effects is judged by considering the value individuals place upon the main outcomes, the degree of desirable and undesirable effects, and the certainty of those estimates. | The overall balance of effects can be judged as either favoring the intervention or comparison. |
| **Resources Required** | An estimate of the cost of the difference in resource use between the intervention and comparison. | An option with large savings is more likely to receive a strong recommendation. |
| **Certainty of Evidence of Required Resources** | It is determined by the likelihood that the research provides valid evidence of cost differences between the intervention and comparison. | Evidence with higher certainty lends to a strong recommendation. |
| **Cost-Effectiveness** | Determines cost-effectiveness by considering uncertainty about or variability in costs or net benefit, sensitivity analyses, and the reliability and applicability of the economic evaluation. | An option that is more cost-effective is more likely to receive a strong recommendation |
| **Equity** | Likelihood of differences in the relative effectiveness of the intervention for disadvantaged subgroups that influence the absolute effectiveness of the intervention. | An option with a greater likelihood to favor equity is more likely to receive a strong recommendation. |
| **Acceptability** | Likelihood of key stakeholders to accept the distribution of benefits, harms, costs, and ethical concerns associated with the intervention, over an extended period. | An option more acceptable to most stakeholders is more likely to receive a strong recommendation. |
| **Feasibility** | Practicality of sustained use of the intervention. | An option more feasible to most stakeholders is more likely to receive a strong recommendation. |

| **Supplementary Table 2: Dummy Evidence to Decision (ETD) table** | | | |
| --- | --- | --- | --- |
| **Question**: Should *Intervention/Suggested Change* be favored over *Comparison/Current Standard of Practice*? | | | |
| **Criteria** | **Research Evidence** | **Additional Considerations** | **Judgment** |
| **Problem:** Is the problem a priority? |  |  | - No - Probably No - Probably Yes - Yes - Varies - Don’t Know |
| **Desirable Effects:** How substantial are the desirable anticipated effects? |  |  | - Trivial - Small - Moderate - Large - Varies - Don’t Know |
| **Undesirable Effects**: How substantial are the undesirable anticipated effects? |  |  | - Large - Moderate - Small - Trivial - Varies - Don’t Know |
| **Certainty of Evidence**: What is the overall certainty of the evidence of effects? |  |  | - Very Low - Low - Moderate - High - No Included Studies |
| **Value**: Is there important uncertainty about or variability in how much people value the main outcomes? |  |  | - Important Uncertainty or Variability - Possible Uncertainty or Variability - Probably No Important Uncertainty or Variability - No Important Variability or Uncertainty |
| **Balance of Effects**: Does the balance between desirable and undesirable effects favor the intervention or the comparison? |  |  | - Favors Comparison - Probably Favors the Comparison - Does Not Favor Either the Intervention or Comparison - Probably Favors the Intervention - Favors Intervention - Varies - Don’t Know |
| **Resources Required**: How large are the resource requirements (costs)? |  |  | - Large Costs - Moderate Costs - Negligible Costs or Savings - Moderate Savings - Large Savings - Varies - Don’t Know |
| **Certainty of Evidence of Required Resources**: What is the certainty of the evidence of resource requirements (costs)? |  |  | - Very Low - Low - Moderate - High - No Included Studies |
| **Cost-Effectiveness**: Does the cost-effectiveness of the intervention favor the intervention or the comparison? |  |  | - Favors Comparison - Probably Favors the Comparison - Does Not Favor Either the Intervention or Comparison - Probably Favors the Intervention - Favors Intervention - Varies - No Included Studies |
| **Equity**: What would be the impact on health equity? |  |  | - Reduced - Probably Reduced - Probably No Impact - Probably Increased - Increased - Varies - Don’t Know |
| **Acceptability**: Is the intervention acceptable to key stakeholders? |  |  | - No - Probably No - Probably Yes - Yes - Varies - Don’t Know |
| **Feasibility**: Is the intervention feasible to implement? |  |  | - No - Probably No - Probably Yes - Yes - Varies - Don’t Know |
| **Overall Recommendations**   - Strong Recommendation Against the Intervention - Conditional Recommendation Against the Intervention - Conditional Recommendation for Either the Intervention or the Comparison - Conditional Recommendation for the Intervention - Strong Recommendation for the Intervention | | | |

| **Supplementary Table 3: Recommendations adopted with Minor Changes/Additional Comments**  ** Level of Evidence as classified by the source guideline ^(2)^*  *CGM:* Continuous glucose monitoring; *SMBG*: Self-Monitoring of Blood Glucose | | | | |
| --- | --- | --- | --- | --- |
| **#** | **Recommendation** | **Level of Evidence *** | **Reason for Change** | **Revised Recommendation/ “Additional Comment”** |
| **12.16** | **Consider costs of care and insurance coverage rules when developing treatment plans in order to reduce risk of cost-related nonadherence.** | Grade B | Almost 60% of healthcare costs are via out-of-pocket payment by patients ^(3)^. National health coverage is provided to approximately 20% of the population ^(4)^. Third-party payers in the form of private insurance is extremely infrequent ^(5)^. | Consider costs of care when developing treatment plans in order to reduce risk of cost-related nonadherence. |
| **6.3** | **Standardized, single-page glucose reports from CGM devices with visual cues, such as the ambulatory glucose profile (AGP), should be considered as a standard printout for all CGM devices.** | Grade E | Annual income in Pakistan is approximately US$420 on average, with 35% of people living below the international poverty line. The cost of a two-week use of a CGM sensor is approximately $77. Access to health care and pharmacies is limited in rural areas, many of which are without electricity. These factors pose significant barriers to the use of CGM ^(6)^. | “Consider the costs and financial burden associated with CGM devices when incorporating them into diabetes management” |
| **7.11** | **In patients on a multiple daily injection (MDI) or continuous subcutaneous insulin infusion (CSII) regimen, real-time CGM devices should be used as close to daily as possible for maximal benefit.**  **Intermittently scanned CGM devices should be scanned frequently, at a minimum once every 8 hours.** | Grade A |  |  |
| **7.12** | **When used as an adjunct to pre- and postprandial SMBG, CGM can help to achieve A1C targets in diabetes and pregnancy.** | Grade B |  |  |
| **12.5** | **For older adults with type 1 diabetes, CGM should be considered to reduce hypoglycemia.** | Grade **A** |  |  |

| **Supplementary Table 4: Excluded Recommendations**  ** Level of Evidence as classified by the source guideline ^(2)^* | | | |
| --- | --- | --- | --- |
| **#** | **Recommendation** | **Level of Evidence *** | **Reason for Exclusion** |
| **5.5** | **Because DSMES (Diabetes Self-Management, Education and Support) can improve outcomes and reduce costs, reimbursement by third-party payers is recommended.** | Grade B & C | Almost 60% of healthcare costs are via out-of-pocket payment by patients ^(3)^. National health coverage is provided to approximately 20% of the population ^(4)^. Third-party payers in the form of private insurance is extremely infrequent ^(5)^. |
| **12.16** | **Consider insurance coverage rules when developing treatment plans in order to reduce risk of cost-related nonadherence.** | Grade B |  |
| **6.11** | **Glucagon should be prescribed for all individuals at increased risk of level 2 or 3 hypoglycemia so that it is available should it be needed. Caregivers, school personnel, or family members of these individuals should know where it is and when and how to administer it. Glucagon administration is not limited to health care professionals.** | Grade E | Glucagon is not widely available in Pakistan. |
| **10.31** | **In patients with atherosclerotic cardiovascular disease or other cardiovascular (CV) risk factors on a statin with controlled low-density lipoprotein (LDL) cholesterol but elevated triglycerides (135–499 mg/dL), the addition of icosapent ethyl can be considered to reduce CV risk.** | Grade A | Icosapent ethyl is not widely available in Pakistan. |
| **13.110** | **Pediatric diabetes providers should begin to prepare youth for transition to adult health care in early adolescence and, at the latest, at least 1 year before the transition.** | Grade E | Recommendations concerning pediatric patients are not to be included in the Pakistani CPGs for management of T2DM in adults. |
| **13.111** | **Both pediatric and adult diabetes care providers should provide support and resources for transitioning young adults.** | Grade E |  |
| **5.26** | **Children and adolescents with type 1 or type 2 diabetes or prediabetes should engage in 60 minutes/day or more of moderate- or vigorous- intensity aerobic activity, with vigorous muscle- strengthening and bone-strengthening activities at least 3 days/week** | Grade C |  |
| **13.62** | **A reasonable A1C target for most children and adolescents with type 2 diabetes treated with oral agents alone is <7% (53 mmol/mol). More stringent A1C targets (such as <6.5% [48 mmol/mol]) may be appropriate for selected individual patients if they can be achieved without significant hypoglycemia or other adverse effects of treatment. Appropriate patients might include those with short duration of diabetes and lesser degrees of β-cell dysfunction and patients treated with lifestyle or metformin only who achieve significant weight improvement** | Grade E |  |
| **15.1** | **Perform an A1C test on all patients with diabetes or hyperglycemia (blood glucose >140 mg/dL [7.8 mmol/L]) admitted to the hospital if not performed in the prior 3 months.** | Grade B | Recommendations concerning inpatient management are not to be included in the Pakistani CPGs for management of T2DM at a general practitioner level. |
| **15.2** | **Insulin should be administered using validated written or computerized protocols that allow for predefined adjustments in the insulin dosage based on glycemic fluctuations.** | Grade B |  |
| **15.4** | **Insulin therapy should be initiated for treatment of persistent hyperglycemia starting at a threshold ≥180 mg/dL (10.0 mmol/L). Once insulin therapy is started, a target glucose range of 140–180 mg/dL (7.8–10.0 mmol/L) is recommended for the majority of critically ill and noncritically ill patients.** | Grade A |  |
| **15.5** | **More stringent goals, such as 110–140 mg/dL (6.1–7.8 mmol/L), may be appropriate for selected patients if they can be achieved without significant hypoglycemia.** | Grade C |  |
| **15.6** | **Basal insulin or a basal plus bolus correction insulin regimen is the preferred treatment for noncritically ill hospitalized patients with poor oral intake or those who are taking nothing by mouth.** | Grade A |  |
| **15.7** | **An insulin regimen with basal, prandial, and correction components is the preferred treatment for noncritically ill hospitalized patients with good nutritional intake.** | Grade A |  |
| **15.8** | **Use of only a sliding-scale insulin regimen in the inpatient hospital setting is strongly discouraged.** | Grade A |  |
| **15.9** | **A hypoglycemia management protocol should be adopted and implemented by each hospital or hospital system. A plan for preventing and treating hypoglycemia should be established for each patient. Episodes of hypoglycemia in the hospital should be documented in the medical record and tracked.** | Grade E |  |
| **15.10** | **The treatment regimen should be reviewed and changed as necessary to prevent further hypoglycemia when a blood glucose value of <70 mg/dL (3.9 mmol/L) is documented.** | Grade C |  |
| **15.11** | **There should be a structured discharge plan tailored to the individual patient with diabetes.** | Grade B |  |

| **Supplementary Table 5: Summary of Evidence to Decision Table** | | |
| --- | --- | --- |
| **Question***: “Should we recommend screening in persons younger than 45 years vs. above 45 years be used for Diabetes/Prediabetes screening in Pakistan?”* | | |
| **Criteria** | **Research Evidence**  * Added on request for additional information | **Additional Considerations** |
| **Problem** | ● South Asian countries/countries of the Indian Subcontinent have a high prevalence of T2DM in adults (as high as 32.9% in some regions of Pakistan) ^(7-11)^.  ● More than 25% of people with T2DM in Pakistan are aged < 40 years and almost 50% are aged between 40-59 years ^(12)^.  ● Screening in South Asian regions should begin at a younger age (at 30 years) as the prevalence of T2DM amongst people aged < 44 years has increased from 25% to 36% in a 6 year period (2000-2006) ^(13)^.  ● Screening for non-pregnant adults in India should be started at the age of 25 years, particularly in those who are overweight/obese or with a family history of T2DM ^(14)^.  ● Risk-based screening for prediabetes and/or T2DM should be considered after the onset of puberty or after 10 years of age (whichever occurs earlier) in individuals who are overweight/obese or who have one or more risk factor for T2DM ^(15)^. | ● If screening at a younger age is warranted in our population, also consider what the ideal revised age cut-off should be.  ● According to the Pakistan Bureau of Statistics (PBS), > 85% of the population in Pakistan is younger than 45 years and > 70% is younger than 30 years ^(3)^. |
| **Desirable Effects** | ● Early diagnosis of T2DM allowing for earlier implementation of management interventions (self-management education, increasing physical activity, moderate weight reduction, and medications) can substantially control blood glucose levels, reduce complications, delay disease progression, and improve quality of life, even in high-risk patients ^(16-18)^. | Beyond the direct physical benefits of initiating earlier management, also consider what desirable effects an earlier diagnosis could have regarding quality of life and social functionality. |
| **Undesirable Effects** | A mini-systematic review of PubMed & Google Scholar yielded 50 articles, none of which were deemed relevant on title and abstract screening. No evidence regarding undesirable effects was found in the source guideline or local published literature. | - |
| **Value** | ● In low-income settings, individuals present to a healthcare provider only when the symptoms of T2DM are obvious and complications may have set in ^(19)^. | Consider majorly the value attributed to the outcome by patients, as they are the main stakeholder. |
| **Balance of Effects** | - | Please review the above data evidence to form your own judgement about the balance of effects. |
| **Resources Required** | Costs of screening T2DM/prediabetes available in Pakistan are as follows:  ● AKU Lab  1) *Hemoglobin A_1C_*: 2700 PKR (13 USD) per test  2) *Random Glucose*: 550 PKR (3 USD) per test  3) *Fasting Glucose*: 550 PKR (3 USD) per test  4) *Oral Glucose Tolerance Test **: 480 PKR (2 USD) per test    ● External Labs  1) *Hemoglobin A_1C_*: 750-1650 PKR (4-8 USD) per test  2) *Random Glucose*: 140-300 PKR (1-2 USD)  per test  3) *Fasting Glucose*: 140-300 PKR (1-2 USD) per test  4) *Oral Glucose Tolerance Test **: 430-1000 (2-5 USD) per test | - |
| **Certainty of Evidence of Required Resources** | All the above data has been sourced via telephone calls to the laboratories. | - |
| **Cost-Effectiveness** | ● Screening for T2DM, beginning at age 30 or 45 years and independent of risk factors, may be cost-effective ^(15)^.  ● Screening for T2DM and prediabetes can be cost-saving, especially in patients at high-risk ^(17)^.  ● Early prevention and management of diabetes are likely to be a cost-saving opportunity for Bangladesh if effectively screening measures for T2DM are implemented ^(20)^.  ● However, though screening may be cost-effective, evidence is still mixed and warrants additional research to substantiate the claim ^(21)^. | Consider whether the cost-effectiveness would ultimately benefit the patient financially, as most healthcare costs are paid out-of-pocket in Pakistan. |
| **Equity** | A mini-systematic review of PubMed & Google Scholar yielded 40 articles, none of which were deemed relevant on title and abstract screening. No evidence regarding undesirable effects was found in the source guideline or local published literature. However, the following evidence from other countries may be interpreted with caution in our setting:  ● Low socioeconomic status is associated with higher T2DM incidence and inequality of T2DM care in a northeast Asian population, despite universal health coverage ^(22)^.  ● Low socioeconomic status, particularly amongst women, is associated with T2DM ^(23)^.  ● Low socioeconomic status, particularly amongst African-American women, is associated with T2DM ^(24)^. | In Pakistan, groups are marginalized on the basis of any number of characteristics, including socioeconomic status, gender or sex, language, religion or sect, race, and political affiliation. Thus, please consider the potential to introduce a degree much-needed equity in the healthcare provided in Pakistan. |
| **Acceptability** | A mini-systematic review of PubMed & Google Scholar yielded 40 articles, none of which were deemed relevant on title and abstract screening. No evidence regarding undesirable effects was found in the source guideline or local published literature | Please consider that while for physicians the acceptability of the intervention may not be in question, patients may have several reservations regarding the need for earlier testing. These may be related to factors such as:  ● Additional costs  ● Additional travel (particularly for patients in rural settings)  ● Additional time (particularly for those commuting long journeys to testing facilities)  ● Additional responsibility of ensuring the routine testing is conducted in a timely manner  ● Stigma associated with undergoing testing for or receiving a diagnosis of T2DM |
| **Feasibility** | A mini-systematic review of PubMed & Google Scholar yielded 40 articles, none of which were deemed relevant on title and abstract screening. No evidence regarding undesirable effects was found in the source guideline or local published literature | Possible factors to consider in Pakistan:  ● Frequency of testing and visit cost.  ● Majorly out-of-pocket health payments.  ● Lack of patient education and awareness regarding the severity and long-term complications of T2DM.  ● The health system of Pakistan starkly different in rural and urban settings. Rural settings are deprived of screening infrastructure. The intervention must be feasible across all settings. |

**Table of Recommendations**

The American Diabetes Association indicates Level of Evidence using the Letters A, B, C, or E to show the evidence level that supports each recommendation:

A: Clear evidence from well-conducted, generalizable randomized controlled trials that are adequately powered.

B: Supportive evidence from well-conducted cohort studies.

C: Supportive evidence from poorly controlled or uncontrolled studies.

E: Expert consensus or clinical experience.

| **Supplementary Table 6: Table of Recommendations** | | | | |
| --- | --- | --- | --- | --- |
| **#** | **Recommendations** | **Adopt** | **Adapt** | **Exclude** |
| 01 | Align approaches to diabetes management with the Chronic Care Model (CCM). This model emphasizes person-centered team care, integrated long-term treatment approaches to diabetes and comorbidities,  and ongoing collaborative communication and goal setting between all team members. **Grade A** | **X** |  |  |
| 02 | Care systems should facilitate team-based care and utilization of patient registries, decision support tools, and community involvement to meet patient needs. **Grade B** | **X** |  |  |
| 03 | Assess diabetes health care maintenance using reliable and relevant data metrics to improve processes of care and health outcomes, with attention to care costs. **Grade B** | **X** |  |  |
| 04 | Assess food insecurity, housing insecurity/homelessness, financial barriers, and social capital/ social community support and apply that information to treatment decisions. **Grade A** | **X** |  |  |
| 05 | Refer patients to local community resources when available. **Grade B** | **X** |  |  |
| 06 | Provide patients with self-management support from lay health coaches, navigators, or community health workers when available. **Grade A** | **X** |  |  |
| 07 | Screening for prediabetes and type 2 diabetes with an informal assessment of risk factors or validated tools should be considered in asymptomatic adults. **Grade B** | **X** |  |  |
| 08 | Testing for prediabetes and/or type 2 diabetes in asymptomatic people should be considered in adults of any age with overweight (BMI 25–29.9 kg/m^2^ or 23–27.4 kg/m^2^ in Asian Americans) or obesity (BMI ≥ 30 kg/m^2^ or ≥ 27.5 kg/m^2^ in Asian Americans) and who have one or more additional risk factors for diabetes. **Grade B** | **X** |  |  |
| 09 | Testing for prediabetes and/or type 2 diabetes should be considered in women with overweight or obesity planning pregnancy and/or who have one or more additional risk factors for diabetes. **Grade C** | **X** |  |  |
| 10 | For all people, testing should begin at age 45 years. **Grade B** |  | **X** |  |
| 11 | If tests are normal, repeat testing carried out at a minimum of 3-year intervals is reasonable, sooner with symptoms. **Grade C** | **X** |  |  |
| 12 | Risk-based screening for prediabetes and/or type 2 diabetes should be considered after the onset of puberty or after 10 years of age, whichever occurs earlier, in children and adolescents with overweight (BMI ≥ 85th percentile) or obesity (BMI ≥ 95th percentile) and who have additional risk factors for diabetes. **Grade B** | **X** |  |  |
| 13 | At least annual monitoring for the development of type 2 diabetes in those with prediabetes is suggested. **Grade E** | **X** |  |  |
| 14 | Refer patients with prediabetes to an intensive lifestyle behavior change program modeled on the Diabetes Prevention Program (DPP) to achieve and maintain 7% loss of initial body weight and increase moderate-intensity physical activity (such as brisk walking) to at least 150 minutes/week. **Grade A** | **X** |  |  |
| 15 | A variety of eating patterns can be considered to prevent diabetes in individuals with prediabetes. **Grade B** | **X** |  |  |
| 16 | Based on patient preference, certified technology- assisted diabetes prevention programs may be effective in preventing type 2 diabetes and should be considered. **Grade B** | X |  |  |
| 17 | Metformin therapy for prevention of type 2 diabetes should be considered in those with prediabetes especially for those with BMI ≥ 35 kg/m^2^, those aged <60 years, and women with prior GDM. **Grade A** | **X** |  |  |
| 18 | Prediabetes is associated with heightened cardiovascular (CV) risk; therefore, screening for and treatment of modifiable risk factors for cardiovascular disease (CVD) are suggested. **Grade B** | **X** |  |  |
| 19 | A patient-centered communication style that uses person-centered and strength-based language and active listening; elicits patient preferences and beliefs; and assesses literacy, numeracy, and potential barriers to care should be used to optimize patient health outcomes and health-related quality of life. **Grade B** | **X** |  |  |
| 20 | People with diabetes can benefit from a coordinated multidisciplinary team that may draw from certified diabetes care and education specialists (CDCES), primary care providers, subspecialty providers, nurses, dietitians, exercise specialists, pharmacists, dentists, podiatrists, and mental health professionals. **Grade E** | **X** |  |  |
| 21 | A complete medical evaluation should be performed at the initial visit to:  ● Confirm the diagnosis and classify diabetes. **Grade A**  ● Evaluate for diabetes complications and potential comorbid conditions. **Grade A**  ● Review previous treatment and risk factor control in patients with established diabetes. **Grade A**  ● Begin patient engagement in the formulation of a care management plan. **Grade A**  ● Develop a plan for continuing care. **Grade A** | **X** |  |  |
| 22 | A follow-up visit should include most components of the initial comprehensive medical evaluation. (See Table 4.1 in the complete 2021 Standards of Care.) **Grade A** | **X** |  |  |
| 23 | Ongoing management should be guided by the assessment of overall health status, diabetes complications, CV risk (see “The Risk Calculator” in “10. CVD AND RISK MANAGEMENT”), hypoglycemia risk, and shared decision-making to set therapeutic goals. **Grade B** | **X** |  |  |
| 24 | Patients with type 2 diabetes or prediabetes and elevated liver enzymes (ALT) or fatty liver on ultrasound should be evaluated for presence of nonalcoholic steatohepatitis and liver fibrosis. **Grade C** | **X** |  |  |
| 25 | In accordance with the national standards for DSMES (Diabetes Self-Management, Education and Support), all people with diabetes should participate in diabetes self-management education and receive the support needed to facilitate the knowledge, decision- making, and skills mastery necessary for diabetes self-care.  **Grade A** | **X** |  |  |
| 26 | There are four critical times to evaluate the need for diabetes self-management education to promote skills acquisition in support of regimen implementation, MNT, and well-being: at diagnosis, annually and/or when not meeting treatment targets, when complicating factors develop (medical, physical, psychosocial), and when transitions in life and care occur.  **Grade E** | **X** |  |  |
| 27 | Clinical outcomes, health status, and well-being are key goals of DSMES that should be measured as part of routine care. **Grade C** | **X** |  |  |
| 28 | DSMES should be patient centered, may be given in group or individual settings and/or use technology, and should be communicated with the entire diabetes care team. **Grade A** | **X** |  |  |
| 29 | Because DSMES can improve outcomes and reduce costs B, reimbursement by third-party payers is recommended. **Grade C** |  |  | **X** |
| 30 | Some barriers to DSMES access may be mitigated through telemedicine approaches. **Grade B** | **X** |  |  |
| 31 | Children and adolescents with type 1 or type 2 diabetes or prediabetes should engage in 60 minutes/day or more of moderate- or vigorous- intensity aerobic activity, with vigorous muscle- strengthening and bone-strengthening activities at least 3 days/week. **Grade C** | **X** |  |  |
| 32 | Most adults with type 1 and type 2 diabetes should engage in 150 minutes or more of moderate- to vigorous-intensity aerobic activity per week, spread over at least 3 days/week, with no more than 2 consecutive days without activity. Shorter durations (minimum 75 minutes/week) of vigorous- intensity or interval training may be sufficient for younger and more physically fit individuals. **Grade C (T1DM)** **and** **Grade B (T2DM)** | **X** |  |  |
| 33 | Adults with type 1 Cand type 2 diabetes should engage in 2–3 sessions/week of resistance exercise on nonconsecutive days. **Grade C (T1DM) and Grade B (T2DM)** | **X** |  |  |
| 34 | All adults, and particularly those with type 2 diabetes, should decrease the amount of time spent in daily sedentary behavior. B Prolonged sitting should be interrupted every 30 minutes for blood glucose benefits. **Grade C** | **X** |  |  |
| 35 | Flexibility training and balance training are recommended 2–3 times/week for older adults with diabetes. Yoga and tai chi may be included based on individual preferences to increase flexibility, muscular strength, and balance. **Grade C** | **X** |  |  |
| 36 | Evaluate baseline physical activity and sedentary time. Promote increase in non-sedentary activities above baseline for sedentary individuals with type 1 Band type 2 diabetes. Examples include walking, yoga, housework, gardening, swimming, and dancing. **Grade C (T1DM) and Grade B (T2DM)** | **X** |  |  |
| 37 | Advise all patients not to use cigarettes and other tobacco products A or e-cigarettes. **Grade A** | **X** |  |  |
| 38 | After identification of tobacco or e-cigarette use, include smoking cessation counseling and other forms of treatment as a routine component of diabetes care. **Grade A** | **X** |  |  |
| 39 | Psychosocial care should be integrated with a collaborative, patient-centered approach and provided to all people with diabetes, with the goals of optimizing health outcomes and health-related quality of life. **Grade A** | **X** |  |  |
| 40 | Psychosocial screening and follow-up may include, but are not limited to, attitudes about diabetes, expectations for medical management and out- comes, affect or mood, general and diabetes-related quality of life, available resources (financial, social, and emotional), and psychiatric history. **Grade E** | **X** |  |  |
| 41 | Providers should consider assessment for symptoms of diabetes distress, depression, anxiety, disordered eating, and cognitive capacities using appropriate standardized and validated tools at the initial visit, at periodic intervals, and when there is a change in disease, treatment, or life circumstance. Including caregivers and family members in this assessment is recommended. **Grade B** | **X** |  |  |
| 42 | Consider screening older adults (aged ⊇65 years) with diabetes for cognitive impairment and depression. **Grade B** | **X** |  |  |
| 43 | Routinely monitor people with diabetes for diabetes distress, particularly when treatment targets are not met and/or at the onset of diabetes complications. **Grade B** | **X** |  |  |
| 44 | Assess glycemic status (A1C or other glycemic measurement) at least two times a year in patients who are meeting treatment goals (and who have stable glycemic control). **Grade E** | **X** |  |  |
| 45 | Assess glycemic status at least quarterly, and as needed, in patients whose therapy has recently changed and/or who are not meeting glycemic goals. **Grade E** | **X** |  |  |
| 46 | Standardized, single-page glucose reports from CGM devices with visual cues, such as the ambulatory glucose profile (AGP), should be considered as a standard printout for all CGM devices. **Grade E** | **X** |  |  |
| 47 | Time in range (TIR) is associated with the risk of microvascular complications, should be an acceptable end point for clinical trials moving forward, and can be used for assessment of glycemic control. Additionally, time below target (<70 and <54 mg/dL [3.9 and 3.0 mmol/L]) and time above target (>180 mg/dL [10.0 mmol/L]) are useful parameters for reevaluation of the treatment regimen. **Grade C** | **X** |  |  |
| 48 | ● An A1C goal for many nonpregnant adults of <7% (53 mmol/mol) without significant hypoglycemia is appropriate. **Grade A**  ● If using AGP/GMI to assess glycemia, a parallel goal is a TIR of >70% with time below range <4%. **Grade B** | **X** |  |  |
| 49 | On the basis of provider judgment and patient preference, achievement of lower A1C levels than the goal of 7% may be acceptable, and even beneficial, if it can be achieved safely without significant hypoglycemia or other adverse effects of treatment. **Grade C** | **X** |  |  |
| 50 | Less stringent A1C goals (such as <8% [64 mmol/mol]) may be appropriate for patients with limited life expectancy, or where the harms of treatment are greater than the benefits. **Grade B** | **X** |  |  |
| 51 | Occurrence and risk for hypoglycemia should be reviewed at every encounter and investigated as indicated. **Grade C** | **X** |  |  |
| 52 | Glucose (~15–20 g) is the preferred treatment for the conscious individual with blood glucose ,70 mg/dL (3.9 mmol/L), although any form of carbohydrate that contains glucose may be used. Fifteen minutes after treatment, if SMBG shows continued hypoglycemia, the treatment should be repeated. Once the SMBG or glucose pattern is trending up, the individual should consume a meal or snack to prevent recurrence of hypoglycemia. **Grade B** | **X** |  |  |
| 53 | Glucagon should be prescribed for all individuals at increased risk of level 2 or 3 hypoglycemia so that it is available should it be needed. Caregivers, school personnel, or family members of these individuals should know where it is and when and how to administer it. Glucagon administration is not limited to health care professionals. **Grade E** |  |  | **X** |
| 54 | Hypoglycemia unawareness or one or more episodes of level 3 hypoglycemia should trigger hypoglycemia avoidance education and reevaluation of the treatment regimen. **Grade E** | **X** |  |  |
| 55 | Insulin-treated patients with hypoglycemia unawareness, one level 3 hypoglycemic event, or a pattern of unexplained level 2 hypoglycemia should be advised to raise their glycemic targets to strictly avoid hypoglycemia for at least several weeks in order to partially reverse hypoglycemia unaware- ness and reduce risk of future episodes. **Grade A** | **X** |  |  |
| 56 | Ongoing assessment of cognitive function is suggested with increased vigilance for hypoglycemia by the clinician, patient, and caregivers if low cognition or declining cognition is found. **Grade B** | **X** |  |  |
| 57 | Use of technology should be individualized based on a patient’s needs, desires, skill level, and availability of devices. **Grade E** | **X** |  |  |
| 58 | People who are on insulin using SMBG should be encouraged to test when appropriate based on their insulin regimen. This may include testing when fasting, prior to meals and snacks, at bedtime, prior to exercise, when low blood glucose is suspected, after treating low blood glucose until they are normoglycemic, and prior to and while performing critical tasks such as driving. **Grade B** | **X** |  |  |
| 59 | When prescribed as part of a DSMES program, SMBG may help to guide treatment decisions  and/or self- management for patients taking less frequent insulin injections. **Grade B** | **X** |  |  |
| 60 | In patients on a multiple daily injection (MDI) or continuous subcutaneous insulin infusion (CSII) regimen, real-time CGM devices should be used as close to daily as possible for maximal benefit.  Intermittently scanned CGM devices should be scanned frequently, at a minimum once every 8 hours. **Grade A** | **X** |  |  |
| 61 | When used as an adjunct to pre- and postprandial SMBG, CGM can help to achieve A1C targets in diabetes and pregnancy.  **Grade B** | **X** |  |  |
| 62 | Use patient-centered, nonjudgmental language that fosters collaboration between patients and providers, including people-first language (e.g., “person with obesity” rather than “obese person”). **Grade** **E** | **X** |  |  |
| 63 | Measure height and weight and calculate BMI at annual visits or more frequently. Assess weight trajectory to inform treatment considerations. **Grade** **E** | **X** |  |  |
| 64 | ● Based on clinical considerations, such as the presence of comorbid heart failure (HF) or significant unexplained weight gain or loss, weight may need to be monitored and evaluated more frequently. **Grade B**  ● If deterioration of medical status is associated with significant weight gain or loss, inpatient evaluation should be considered, especially focused on associations between medication use, food intake, and glycemic status. **Grade** **E** | **X** |  |  |
| 65 | Accommodations should be made to provide privacy during weighing. **Grade** **E** | **X** |  |  |
| 66 | Diet, physical activity, and behavioral therapy designed to achieve and maintain ≥5% weight loss is recommended for most patients with type 2 diabetes who have overweight or obesity and are ready to achieve weight loss. Greater benefits in control of diabetes and CV risk may be gained from even greater weight loss. **Grade** **B** | **X** |  |  |
| 67 | Such interventions should include a high frequency of counseling (≥16 sessions in 6 months) and focus on dietary changes, physical activity, and behavioral strategies to achieve a 500–750 kcal/day energy deficit. **Grade A** | **X** |  |  |
| 68 | An individual’s preferences, motivation, and life circumstances should be considered, along with medical status, when weight-loss interventions are recommended. **Grade C** | **X** |  |  |
| 69 | Behavioral changes that create an energy deficit, regardless of macronutrient composition, will result in weight loss. Dietary recommendations should be individualized to the patient’s preferences and nutritional needs. **Grade A** | **X** |  |  |
| 70 | Evaluate systemic, structural, and socioeconomic factors that may impact dietary patterns and food choices, such as food insecurity and hunger, access to healthful food options, cultural circumstances, and SDOH. **Grade C** | **X** |  |  |
| 71 | For patients who achieve short-term weight-loss goals, long-term (≥1 year) weight-maintenance programs are recommended when available. Such programs should, at minimum, provide monthly contact and support, recommend ongoing monitoring of body weight (weekly or more frequently) and other self-monitoring strategies, and encourage high levels of physical activity (200–300 minutes/week). **Grade A** | **X** |  |  |
| 72 | Short-term dietary intervention using structured, very-low-calorie diets (800–1,000 kcal/day) may be prescribed for carefully selected patients by trained practitioners in medical settings with close monitoring. Long-term, comprehensive weight-maintenance strategies and counseling should be integrated to maintain weight loss. **Grade B** | **X** |  |  |
| 73 | When choosing glucose-lowering medications for patients with type 2 diabetes and overweight or obesity, consider the medication's effect on weight. **Grade B** | **X** |  |  |
| 74 | Whenever possible, minimize medications for comorbid conditions that are associated with weight gain. **Grade E** | **X** |  |  |
| 75 | Weight-loss medications are effective as adjuncts to diet, physical activity, and behavioral counseling for selected patients with type 2 diabetes and BMI ≥27 kg/m^2^. Potential benefits and risks must be considered. **Grade A** | **X** |  |  |
| 76 | If a patient’s response to weight-loss medication is effective (typically defined as >5% weight loss after 3 months’ use), further weight loss is likely with continued use. When early response is insufficient (typically <5% weight loss after 3 months’ use), or if there are significant safety or tolerability issues, consider discontinuation of the medication and evaluate alternative medications or treatment approaches. **Grade A** | **X** |  |  |
| 77 | Metabolic surgery should be a recommended option to treat type 2 diabetes in screened surgical candidates with BMI ≥40 kg/m^2^ (BMI ≥37.5 kg/m^2^ in Asian Americans) and in adults with BMI 35.0–39.9 kg/m^2^ (32.5–37.4 kg/m^2^ in Asian Americans) who do not achieve durable weight loss and improvement in comorbidities (including hyperglycemia) with nonsurgical methods. **Grade A** | **X** |  |  |
| 78 | Metabolic surgery may be considered as an option to treat type 2 diabetes in adults with BMI 30.0–34.9 kg/m^2^ (27.5–32.4 kg/m^2^ in Asian Americans) who do not achieve durable weight loss and improvement in comorbidities (including hyperglycemia) with nonsurgical methods. **Grade A** | **X** |  |  |
| 79 | Metabolic surgery should be performed in high-volume centers with multidisciplinary teams knowledgeable about and experienced in the management of diabetes and gastrointestinal surgery. **Grade E** | **X** |  |  |
| 80 | Long-term lifestyle support and routine monitoring of micronutrient and nutritional status must be provided to patients after surgery, according to guidelines for postoperative management of metabolic surgery by national and international professional societies. **Grade C** | **X** |  |  |
| 81 | People being considered for metabolic surgery should be evaluated for comorbid psychological conditions and social and situational circumstances that have the potential to interfere with surgery outcomes. **Grade** **B** | **X** |  |  |
| 82 | People who undergo metabolic surgery should routinely be evaluated to assess the need for ongoing mental health services to help with the adjustment to medical and psychosocial changes after surgery. **Grade C** | **X** |  |  |
| 83 | Most people with type 1 diabetes should be treated with MDI of prandial and basal insulin or CSII. **Grade A** | **X** |  |  |
| 84 | Most individuals with type 1 diabetes should use rapid acting insulin analogs to reduce hypoglycemia risk. **Grade A** | **X** |  |  |
| 85 | Patients with type 1 diabetes should receive education on how to match prandial insulin doses to carbohydrate intake, premeal blood glucose, and anticipated physical activity. **Grade C** | **X** |  |  |
| 86 | Metformin is the preferred initial pharmacologic agent for the treatment of type 2 diabetes. **Grade A** | **X** |  |  |
| 87 | Once initiated, metformin should be continued as long as it is tolerated and not contraindicated; other agents, including insulin, should be added to metformin. **Grade A** | **X** |  |  |
| 88 | Early combination therapy can be considered in some patients at treatment initiation to extend the time to treatment failure. **Grade A** | **X** |  |  |
| 89 | The early introduction of insulin should be considered if there is evidence of ongoing catabolism (weight loss), if symptoms of hyperglycemia are present, or when A1C levels (>10% [86 mmol/mol]) or blood glucose levels (≥300 mg/dL [16.7 mmol/L]) are very high. **Grade E** | **X** |  |  |
| 90 | A patient-centered approach should be used to guide the choice of pharmacologic agents. Considerations include effect on CV and renal comorbidities, efficacy, hypoglycemia risk, impact on weight, cost, risk for side effects, and patient preferences. **Grade E** | **X** |  |  |
| 91 | Among patients with type 2 diabetes who have established atherosclerotic CVD (ASCVD) or indicators of high risk, established kidney disease, or HF, a sodium–glucose cotransporter 2 (SGLT2) inhibitor or glucagon-like peptide 1 (GLP-1) receptor agonist with demonstrated CVD benefit is recommended as part of the glucose-lowering regimen independent of A1C and in consideration of patient-specific factors. **Grade A** | **X** |  |  |
| 92 | In patients with type 2 diabetes, a GLP-1 receptor agonist is preferred to insulin when possible. **Grade A** | **X** |  |  |
| 93 | Recommendation for treatment intensification for patients not meeting treatment goals should not be delayed. **Grade A** | **X** |  |  |
| 94 | The medication regimen and medication-taking behavior should be reevaluated at regular intervals (every 3–6 months) and adjusted as needed to incorporate specific factors that impact choice of treatment. **Grade E** | **X** |  |  |
| 95 | Clinicians should be aware of the potential for over-basalization with insulin therapy. Clinical signals that may prompt evaluation of over-basalization include basal dose more than ∼0.5 IU/kg, high bedtime-morning or post-pre-prandial glucose differential, hypoglycemia (aware or unaware), and high variability. Indication of over-basalization should prompt reevaluation to further individualize therapy. **Grade E** | **X** |  |  |
| 96 | Blood pressure should be measured at every routine clinical visit. Patients found to have elevated blood pressure (≥140/90 mmHg) should have blood pressure confirmed using multiple readings, including measurements on a separate day, to diagnose hypertension. **Grade B** | **X** |  |  |
| 97 | All hypertensive patients with diabetes should monitor their blood pressure at home. **Grade B** | **X** |  |  |
| 98 | For patients with diabetes and hypertension, blood pressure targets should be individualized through a shared decision-making process that addresses CV risk, potential adverse effects of antihypertensive medications, and patient preferences. **Grade C** | **X** |  |  |
| 99 | For individuals with diabetes and hypertension at higher CV risk (existing ASCVD or 10-year ASCVD risk ≥15%), a blood pressure target of <130/80 mmHg may be appropriate if it can be safely attained. **Grade C** | **X** |  |  |
| 100 | For individuals with diabetes and hypertension at lower risk for CVD (10-year ASCVD risk <15%), treat to a blood pressure target of <140/90 mmHg. **Grade** **A** | **X** |  |  |
| 101 | For patients with blood pressure >120/80 mmHg, lifestyle intervention consists of weight loss when indicated, a Dietary Approaches to Stop Hypertension (DASH)-style eating pattern including reducing sodium and increasing potassium intake, moderation of alcohol intake, and increased physical activity. **Grade A** | **X** |  |  |
| 102 | Patients with confirmed office-based blood pressure ≥140/90 mmHg should, in addition to lifestyle therapy, have prompt initiation and timely titration of pharmacologic therapy to achieve blood pressure goals. **Grade A** | **X** |  |  |
| 103 | Patients with confirmed office-based blood pressure ≥160/100 mmHg should, in addition to lifestyle therapy, have prompt initiation and timely titration of two drugs or a single-pill combination of drugs demonstrated to reduce CV events in patients with diabetes. **Grade A** | **X** |  |  |
| 104 | ● Treatment for hypertension should include drug classes demonstrated to reduce CV events in patients with diabetes. **Grade A**  ● ACE inhibitors or angiotensin receptor blockers (ARBs) are recommended first-line therapy for hypertension in people with diabetes and coronary artery disease (CAD). **Grade A** | **X** |  |  |
| 105 | Multiple-drug therapy is generally required to achieve blood pressure targets. However, combinations of ACE inhibitors and ARBs and combinations of ACE inhibitors or ARBs with direct renin inhibitors should not be used. **Grade A** | **X** |  |  |
| 106 | ● An ACE inhibitor or ARB, at the maximum tolerated dose indicated for blood pressure treatment, is the recommended first-line treatment for hypertension in patients with diabetes and urinary albumin-to-creatinine ratio (UACR) ≥300 mg/g creatinine (**Grade A**) or 30–299 mg/g creatinine (**Grade B).**  ● If one class is not tolerated, the other should be substituted. **Grade B** | **X** |  |  |
| 107 | For patients treated with an ACE inhibitor, ARB, or diuretic, serum creatinine/estimated glomerular filtration rate (eGFR) and serum potassium levels should be monitored at least annually. **Grade B** | **X** |  |  |
| 108 | Patients with hypertension who are not meeting blood pressure targets on three classes of antihypertensive medications (including a diuretic) should be considered for mineralocorticoid receptor antagonist therapy. **Grade B** | **X** |  |  |
|  | Lifestyle modification focusing on weight loss (if indicated); application of a Mediterranean style or DASH eating pattern; reduction of saturated fat and trans fat; increase of dietary n-3 fatty acids, viscous fiber, and plant stanols/sterols intake; and increased physical activity should be recommended to improve the lipid profile and reduce the risk of developing ASCVD in patients with diabetes. **Grade A** | **X** |  |  |
| 110 | Intensify lifestyle therapy and optimize glycemic control for patients with elevated triglyceride levels (≥150 mg/dL [1.7 mmol/L]) and/or low HDL cholesterol (<40 mg/dL [1.0 mmol/L] for men, <50 mg/dL [1.3 mmol/L] for women). **Grade C** | **X** |  |  |
| 111 | In adults not taking statins or other lipid-lowering therapy, it is reasonable to obtain a lipid profile at the time of diabetes diagnosis, at an initial medical evaluation, and every 5 years thereafter if under the age of 40 years, or more frequently if indicated. **Grade E** | **X** |  |  |
| 112 | Obtain a lipid profile at initiation of statins or other lipid-lowering therapy, 4–12 weeks after initiation or a change in dose, and annually thereafter as it may help to monitor the response to therapy and inform medication adherence. **Grade E** | **X** |  |  |
| 113 | For patients with diabetes aged 40–75 years without ASCVD, use moderate-intensity statin therapy in addition to lifestyle therapy. **Grade A** | **X** |  |  |
| 114 | For patients with diabetes aged 20–39 years with additional ASCVD risk factors, it may be reasonable to initiate statin therapy in addition to lifestyle therapy. **Grade C** | **X** |  |  |
| 115 | In patients with diabetes at higher risk, especially those with multiple ASCVD risk factors or aged 50–70 years, it is reasonable to use high-intensity statin therapy. **Grade B** | **X** |  |  |
| 116 | In adults with diabetes and 10-year ASCVD risk of 20% or higher, it may be reasonable to add ezetimibe to maximally tolerated statin therapy to reduce LDL cholesterol levels by 50% or more. **Grade C** | **X** |  |  |
| 117 | For patients of all ages with diabetes and ASCVD, high-intensity statin therapy should be added to lifestyle therapy. **Grade A** | **X** |  |  |
| 118 | For patients with diabetes and ASCVD considered very high risk using specific criteria, if LDL cholesterol is ≥70 mg/dL on maximally tolerated statin dose, consider adding additional LDL-lowering therapy (such as ezetimibe or PCSK9 inhibitor). **Grade A**. Ezetimibe may be preferred due to lower cost. | **X** |  |  |
| 119 | For patients who do not tolerate the intended intensity, the maximally tolerated statin dose should be used. **Grade E** | **X** |  |  |
| 120 | In adults with diabetes aged >75 years already on statin therapy, it is reasonable to continue statin treatment. **Grade B** | **X** |  |  |
| 121 | In adults with diabetes aged >75 years, it may be reasonable to initiate statin therapy after discussion of potential benefits and risks. **Grade C** | **X** |  |  |
| 122 | Statin therapy is contraindicated in pregnancy. **Grade B** | **X** |  |  |
| 123 | For patients with fasting triglyceride levels ≥500 mg/dL, evaluate for secondary causes of hypertriglyceridemia and consider medical therapy to reduce the risk of pancreatitis. **Grade C** | **X** |  |  |
| 124 | In adults with moderate hypertriglyceridemia (fasting or non-fasting triglycerides 175–499 mg/dL), clinicians should address and treat lifestyle factors (obesity and metabolic syndrome), secondary factors (diabetes, chronic liver or kidney disease and/or nephrotic syndrome, hypothyroidism), and medications that raise triglycerides. **Grade C** | **X** |  |  |
| 125 | In patients with ASCVD or other CV risk factors on a statin with controlled LDL cholesterol but elevated triglycerides (135–499 mg/dL), the addition of icosapent ethyl can be considered to reduce CV risk. **Grade A** |  |  | X |
| 126 | Statin plus fibrate combination therapy has not been shown to improve ASCVD outcomes and is generally not recommended. **Grade A** | **X** |  |  |
| 127 | Statin plus niacin combination therapy has not been shown to provide additional CV benefit above statin therapy alone, may increase the risk of stroke with additional side effects, and is generally not recommended. **Grade A** | **X** |  |  |
| 128 | Use aspirin therapy (75–162 mg/day) as a secondary prevention strategy in those with diabetes and a history of ASCVD. **Grade A** | **X** |  |  |
| 129 | For patients with ASCVD and documented aspirin allergy, clopidogrel (75 mg/day) should be used. **Grade B** | **X** |  |  |
| 130 | Dual antiplatelet therapy (with low-dose aspirin and a P2Y12 inhibitor) is reasonable for a year after an acute coronary syndrome and may have benefits beyond this period. **Grade A** | **X** |  |  |
| 131 | Long-term treatment with dual antiplatelet therapy should be considered for patients with prior coronary intervention, high ischemic risk, and low bleeding risk to prevent major adverse CV events (MACE). **Grade A** | **X** |  |  |
| 132 | Combination therapy with aspirin plus low-dose rivaroxaban should be considered for patients with stable coronary and/or PAD and low bleeding risk to prevent major adverse limb and CV events. **Grade A** | **X** |  |  |
| 133 | Aspirin therapy (75–162 mg/day) may be considered as a primary prevention strategy in those with diabetes who are at increased CV risk, after a comprehensive discussion with the patient on the benefits versus the comparable increased risk of bleeding. **Grade A** | **X** |  |  |
| 134 | In asymptomatic patients, routine screening for CAD is not recommended, as it does not improve outcomes as long as ASCVD risk factors are treated. **Grade A** | **X** |  |  |
| 135 | Consider investigations for CAD in the presence of any of the following: atypical cardiac symptoms (e.g., unexplained dyspnea, chest discomfort); signs or symptoms of associated vascular disease, including carotid bruits, transient ischemic attack, stroke, claudication, or PAD; or electrocardiogram abnormalities (e.g., Q waves). **Grade E** | **X** |  |  |
| 136 | Among patients with type 2 diabetes who have established ASCVD or established kidney disease, an SGLT2 inhibitor or GLP-1 receptor agonist with demonstrated CVD benefit (see Tables 10.3B and 10.3C in the complete 2021 Standards of Care) is recommended as part of the comprehensive CV risk reduction and/or glucose-lowering regimens. **Grade A** | **X** |  |  |
| 137 | In patients with type 2 diabetes and established ASCVD, multiple ASCVD risk factors, or DKD, an SGLT2 inhibitor with demonstrated CV benefit is recommended to reduce the risk of MACE and/or HF hospitalization. **Grade A** | X |  |  |
| 138 | In patients with type 2 diabetes and established ASCVD or multiple risk factors for ASCVD, a GLP-1 receptor agonist with demonstrated CV benefit is recommended to reduce the risk of MACE. **Grade A** | **X** |  |  |
| 139 | In patients with type 2 diabetes and established HF with reduced ejection fraction (HFrEF), an SGLT2 inhibitor with proven benefit in this patient population is recommended to reduce risk of worsening HF and CV death. **Grade A** | **X** |  |  |
| 140 | In patients with known ASCVD, particularly CAD, ACE inhibitor or ARB therapy is recommended to reduce the risk of CV events. **Grade A** | **X** |  |  |
| 141 | In patients with prior MI, β-blockers should be continued for 3 years after the event. **Grade B** | **X** |  |  |
| 142 | Treatment of patients with (HFrEF) should include a β-blocker with proven CV outcomes benefit, unless otherwise contraindicated. **Grade A** | **X** |  |  |
| 143 | In patients with type 2 diabetes with stable HF, metformin may be continued for glucose lowering if eGFR remains >30 mL/min/1.73 m^2^ but should be avoided in unstable or hospitalized patients with HF. **Grade B** | **X** |  |  |
| 144 | At least annually, urinary albumin (e.g., spot UACR) and eGFR should be assessed in patients with type 1 diabetes with duration of ≥5 years and in all patients with type 2 diabetes regardless of treatment. **Grade B** | **X** |  |  |
| 145 | Patients with diabetes and urinary albumin >300 mg/g creatinine and/or an eGFR 30–60 mL/min/1.73 m^2^ should be monitored twice annually to guide therapy. **Grade B** | **X** |  |  |
| 146 | Optimize glucose control to reduce the risk or slow the progression of CKD. **Grade A** | **X** |  |  |
| 147 | For patients with type 2 diabetes and DKD, consider use of an SGLT2 inhibitor in patients with an eGFR ≥30 mL/min/1.73 m^2^ and urinary albumin >300 mg/g creatinine. **Grade A** | **X** |  |  |
| 148 | In patients with type 2 diabetes and DKD, consider use of SGLT2 inhibitors additionally for CV risk reduction when eGFR and urinary albumin creatinine are ≥30 mL/min/1.73 m^2^ or >300 mg/g, respectively. **Grade A** | **X** |  |  |
| 149 | In patients with CKD who are at increased risk for CV events, use of a GLP-1 receptor agonist reduces renal end points, primarily albuminuria, progression of albuminuria, and CV events ([Table 9.1](https://clinical.diabetesjournals.org/content/39/1/14#T7)). **Grade A** | **X** |  |  |
| 150 | Optimize blood pressure control to reduce the risk or slow the progression of CKD. **Grade A** | **X** |  |  |
| 151 | Do not discontinue renin-angiotensin system blockade for minor increases in serum creatinine (<30%) in the absence of volume depletion. **Grade A** | **X** |  |  |
| 152 | For people with non-dialysis-dependent CKD, dietary protein intake should be ∼0.8 g/kg body weight per day (the recommended daily allowance). **A** For patients on dialysis, higher levels of dietary protein intake should be considered since malnutrition is a major problem in some dialysis patients. **Grade B** | **X** |  |  |
| 153 | In nonpregnant patients with diabetes and hypertension, either an ACE inhibitor or an ARB is recommended for those with modestly elevated UACR (30–299 mg/g creatinine) (**Grade B)** and is strongly recommended for those with UACR ≥300 mg/g creatinine and/or eGFR <60 mL/min/1.73 m^2^ (**Grade A)** | **X** |  |  |
| 154 | Periodically monitor serum creatinine and potassium levels for the development of increased creatinine or changes in potassium when ACE inhibitors, ARBs, or diuretics are used. **Grade B** | **X** |  |  |
| 155 | An ACE inhibitor or an ARB is not recommended for the primary prevention of CKD in patients with diabetes who have normal blood pressure, normal UACR (<30 mg/g creatinine), and normal eGFR. **Grade A** | **X** |  |  |
| 156 | Patients should be referred for evaluation by a nephrologist if they have an eGFR <30 mL/min/1.73 m^2^. **Grade A** | **X** |  |  |
| 157 | Promptly refer to a physician experienced in the care of kidney disease for uncertainty about the etiology of kidney disease, difficult management issues, and rapidly progressing kidney disease. **Grade A** | **X** |  |  |
| 158 | Optimize glycemic control to reduce the risk or slow the progression of diabetic retinopathy. **Grade A** | **X** |  |  |
| 159 | Optimize blood pressure and serum lipid control to reduce the risk or slow the progression of diabetic retinopathy. **Grade A** | **X** |  |  |
| 160 | Adults with type 1 diabetes should have an initial dilated and comprehensive eye examination by an ophthalmologist or optometrist within 5 years after the onset of diabetes. **Grade B** | **X** |  |  |
| 161 | Patients with type 2 diabetes should have an initial dilated and comprehensive eye examination by an ophthalmologist or optometrist at the time of the diabetes diagnosis. **Grade B** | **X** |  |  |
| 162 | If there is no evidence of retinopathy for one or more annual eye exams and glycemia is well controlled, then screening every 1–2 years may be considered. If any level of diabetic retinopathy is present, subsequent dilated retinal examinations should be repeated at least annually by an ophthalmologist or optometrist. If retinopathy is progressing or sight-threatening, then examinations will be required more frequently. **Grade B** | **X** |  |  |
| 163 | Programs that use retinal photography (with remote reading or use of a validated assessment tool) to improve access to diabetic retinopathy screening can be appropriate screening strategies for diabetic retinopathy. Such programs need to provide pathways for timely referral for a comprehensive eye examination when indicated. **Grade B** | **X** |  |  |
| 164 | Women with preexisting type 1 or type 2 diabetes who are planning pregnancy or who are pregnant should be counseled on the risk of development and/or progression of diabetic retinopathy. **Grade** **B** | **X** |  |  |
| 165 | Eye examinations should occur before pregnancy or in the first trimester in patients with preexisting type 1 or type 2 diabetes, and then patients should be monitored every trimester and for 1 year postpartum as indicated by the degree of retinopathy. **Grade B** | **X** |  |  |
| 166 | Promptly refer patients with any level of macular edema, severe non-proliferative diabetic retinopathy (a precursor of proliferative diabetic retinopathy), or any proliferative diabetic retinopathy to an ophthalmologist who is knowledgeable and experienced in the management of diabetic retinopathy. **Grade A** | **X** |  |  |
| 167 | The traditional standard treatment, pan retinal laser photocoagulation therapy, is indicated to reduce the risk of vision loss in patients with high-risk proliferative diabetic retinopathy and, in some cases, severe non-proliferative diabetic retinopathy. **Grade A** | **X** |  |  |
| 168 | The presence of retinopathy is not a contraindication to aspirin therapy for cardio protection, as aspirin does not increase the risk of retinal hemorrhage. **Grade A** | **X** |  |  |
| 169 | All patients should be assessed for diabetic peripheral neuropathy starting at diagnosis of type 2 diabetes and 5 years after the diagnosis of type 1 diabetes and at least annually thereafter. **Grade B** | **X** |  |  |
| 170 | Assessment for distal symmetric polyneuropathy should include a careful history and assessment of either temperature or pinprick sensation (small fiber function) and vibration sensation using a 128-Hz tuning fork (for large-fiber function). All patients should have annual 10-g monofilament testing to identify feet at risk for ulceration and amputation. **Grade B** | **X** |  |  |
| 171 | Symptoms and signs of autonomic neuropathy should be assessed in patients with microvascular complications. **Grade E** | **X** |  |  |
| 172 | Optimize glucose control to prevent or delay the development of neuropathy in patients with type 1 diabetes **A** and to slow the progression of neuropathy in patients with type 2 diabetes. **Grade B** | **X** |  |  |
| 173 | Assess and treat patients to reduce pain related to diabetic peripheral neuropathy (**Grade** **B)** and symptoms of autonomic neuropathy and to improve quality of life (**Grade E).** | **X** |  |  |
| 174 | Pregabalin, duloxetine, or gabapentin are recommended as initial pharmacologic treatments for neuropathic pain in diabetes. **Grade A** | **X** |  |  |
| 175 | Perform a comprehensive foot evaluation at least annually to identify risk factors for ulcers and amputations. **Grade B** | **X** |  |  |
| 176 | Patients with evidence of sensory loss or prior ulceration or amputation should have their feet inspected at every visit. **Grade B** | **X** |  |  |
| 177 | Obtain a prior history of ulceration, amputation, Charcot foot, angioplasty or vascular surgery, cigarette smoking, retinopathy, and renal disease and assess current symptoms of neuropathy (pain, burning, numbness) and vascular disease (leg fatigue, claudication). **Grade B** | **X** |  |  |
| 178 | The examination should include inspection of the skin, assessment of foot deformities, neurological assessment (10-g monofilament testing with at least one other assessment: pinprick, temperature, vibration), and vascular assessment including pulses in the legs and feet. **Grade B** | **X** |  |  |
| 179 | Patients with symptoms of claudication or decreased or absent pedal pulses should be referred for ankle-brachial index and for further vascular assessment as appropriate. **Grade C** | **X** |  |  |
| 180 | A multidisciplinary approach is recommended for individuals with foot ulcers and high-risk feet (e.g., dialysis patients and those with Charcot foot or prior ulcers or amputation). **Grade B** | **X** |  |  |
| 181 | Refer patients who smoke or who have histories of prior lower-extremity complications, loss of protective sensation, structural abnormalities, or PAD to foot care specialists for ongoing preventive care and lifelong surveillance. **Grade C** | **X** |  |  |
| 182 | Provide general preventive foot self-care education to all patients with diabetes. **Grade B** | **X** |  |  |
| 183 | The use of specialized therapeutic footwear is recommended for high-risk patients with diabetes, including those with severe neuropathy, foot deformities, ulcers, callous formation, poor peripheral circulation, or history of amputation. **Grade B** | **X** |  |  |
| 184 | Consider the assessment of medical, psychological, functional (self-management abilities), and social geriatric domains in older adults to provide a framework to determine targets and therapeutic approaches for diabetes management. **Grade B** | **X** |  |  |
| 185 | Screen for geriatric syndromes (i.e., polypharmacy, cognitive impairment, depression, urinary incontinence, falls, and persistent pain) in older adults, as they may affect diabetes self-management and diminish quality of life. **Grade B** | **X** |  |  |
| 186 | Screening for early detection of mild cognitive impairment or dementia should be performed for adults 65 years of age or older at the initial visit and annually as appropriate. **Grade B** | X |  |  |
| 187 | Because older adults with diabetes have a greater risk of hypoglycemia than younger adults, episodes of hypoglycemia should be ascertained and addressed at routine visits. **Grade B** | **X** |  |  |
| 188 | For older adults with type 1 diabetes, CGM should be considered to reduce hypoglycemia. **Grade A** | **X** |  |  |
| 189 | Older adults who are otherwise healthy with few coexisting chronic illnesses and intact cognitive function and functional status should have lower glycemic goals (such as A1C <7.0–7.5% [53–58 mmol/mol]), while those with multiple coexisting chronic illnesses, cognitive impairment, or functional dependence should have less stringent glycemic goals (such as A1C <8.0–8.5% [64–69 mmol/mol]). **Grade C** | **X** |  |  |
| 190 | Glycemic goals for some older adults might reasonably be relaxed as part of individualized care, but hyperglycemia leading to symptoms or risk of acute hyperglycemia complications should be avoided in all patients. **Grade C** | **X** |  |  |
| 191 | Screening for diabetes complications should be individualized in older adults. Particular attention should be paid to complications that would lead to functional impairment. **Grade C** | **X** |  |  |
| 192 | Treatment of hypertension to individualized target levels is indicated in most older adults. **Grade C** | **X** |  |  |
| 193 | Treatment of other CV risk factors should be individualized in older adults considering the time frame of benefit. Lipid-lowering therapy and aspirin therapy may benefit those with life expectancies at least equal to the time frame of primary prevention or secondary intervention trials. **Grade E** | **X** |  |  |
| 194 | Optimal nutrition and protein intake is recommended for older adults; regular exercise, including aerobic activity, weight-bearing exercise, and/or resistance training, should be encouraged in all older adults who can safely engage in such activities. **Grade B** | **X** |  |  |
| 195 | For older adults with type 2 diabetes, overweight/obesity, and capacity to safely exercise, an intensive lifestyle intervention focused on dietary changes, physical activity, and modest weight loss (e.g., 5–7%) should be considered for its benefits on quality of life, mobility and physical functioning, and cardiometabolic risk factor control. **Grade A** | **X** |  |  |
| 196 | In older adults with type 2 diabetes at increased risk of hypoglycemia, medication classes with low risk of hypoglycemia are preferred. **Grade B** | **X** |  |  |
| 197 | Overtreatment of diabetes is common in older adults and should be avoided. **Grade B** | **X** |  |  |
| 198 | Deintensification (or simplification) of complex regimens is recommended to reduce the risk of hypoglycemia and polypharmacy if it can be achieved within the individualized A1C target. **Grade B** | **X** |  |  |
| 199 | Consider costs of care and insurance coverage rules when developing treatment plans in order to reduce risk of cost-related nonadherence. **Grade B** | **X** |  | **X** |
| 200 | Consider diabetes education for the staff of long-term care (LTC) and rehabilitation facilities to improve the management of older adults with diabetes. **Grade E** | X |  |  |
| 201 | Patients with diabetes residing in LTC facilities need careful assessment to establish individualized glycemic goals and to make appropriate choices of glucose-lowering agents based on their clinical and functional status. **Grade E** | **X** |  |  |
| 202 | When palliative care is needed in older adults with diabetes, providers should initiate conversations regarding the goals and intensity of care.  ● Strict glucose and blood pressure control may not be necessary (**Grade E)**, and reduction of therapy may be appropriate.  ● Similarly, the intensity of lipid management can be relaxed, and withdrawal of lipid-lowering therapy may be appropriate. **Grade A** | **X** |  |  |
| 203 | Overall comfort, prevention of distressing symptoms, and preservation of quality of life and dignity are primary goals for diabetes management at the end of life. **Grade C** | **X** |  |  |
| 204 | A reasonable A1C target for most children and adolescents with type 2 diabetes treated with oral agents alone is <7% (53 mmol/mol). More stringent A1C targets (such as <6.5% [48 mmol/mol]) may be appropriate for selected individual patients if they can be achieved without significant hypoglycemia or other adverse effects of treatment. Appropriate patients might include those with short duration of diabetes and lesser degrees of β-cell dysfunction and patients treated with lifestyle or metformin only who achieve significant weight improvement. **Grade E** | **X** |  |  |
| 205 | Less stringent A1C goals (such as 7.5% [58 mmol/mol]) may be appropriate if there is increased risk of hypoglycemia. **Grade E** | **X** |  |  |
| 206 | Initiate pharmacologic therapy, in addition to behavioral counseling for healthful nutrition and physical activity changes, at diagnosis of type 2 diabetes. **Grade A** | **X** |  |  |
| 207 | In incidentally diagnosed or metabolically stable patients (A1C <8.5% [69 mmol/mol] and asymptomatic), metformin is the initial pharmacologic treatment of choice if renal function is normal. **Grade A** | **X** |  |  |
| 208 | Youth with marked hyperglycemia (blood glucose ≥250 mg/dL [13.9 mmol/L], A1C ≥8.5% [69 mmol/mol]) without acidosis at diagnosis who are symptomatic with polyuria, polydipsia, nocturia, and/or weight loss should be treated initially with basal insulin while metformin is initiated and titrated. **Grade B** | **X** |  |  |
| 209 | If glycemic targets are no longer met with metformin (with or without basal insulin), liraglutide (a GLP-1 receptor agonist) therapy should be considered in children 10 years of age or older if they have no past medical history or family history of medullary thyroid carcinoma or multiple endocrine neoplasia type 2. **Grade A** | **X** |  |  |
| 210 | Patients treated with basal insulin who do not meet glycemic target should be moved to MDI with basal and premeal bolus insulins. **Grade E** | **X** |  |  |
| 211 | Use of medications not approved by the FDA for youth with type 2 diabetes is not recommended outside of research trials. **Grade B** | **X** |  |  |
| 212 | Pediatric diabetes providers should begin to prepare youth for transition to adult health care in early adolescence and, at the latest, at least 1 year before the transition. **Grade E** |  |  | **X** |
| 213 | Both pediatric and adult diabetes care providers should provide support and resources for transitioning young adults. **Grade E** |  |  | **X** |
| 214 | Youth with type 2 diabetes should be transferred to an adult-oriented diabetes specialist when deemed appropriate by the patient and provider. **Grade E** | **X** |  |  |
| 215 | Starting at puberty and continuing in all women with diabetes and reproductive potential, preconception counseling should be incorporated into routine diabetes care. **Grade A** | **X** |  |  |
| 216 | Family planning should be discussed, and effective contraception (with consideration of long-acting, reversible contraception) should be prescribed and used until a woman's treatment regimen and A1C are optimized for pregnancy. **Grade A** | **X** |  |  |
| 217 | Preconception counseling should address the importance of achieving glucose levels as close to normal as is safely possible, ideally A1C <6.5% (48 mmol/mol), to reduce the risk of congenital anomalies, preeclampsia, macrosomia, preterm birth, and other complications. **Grade B** | **X** |  |  |
| 218 | Women with preexisting diabetes who are planning a pregnancy should ideally be managed beginning in preconception in a multidisciplinary clinic including an endocrinologist, maternal-fetal medicine specialist, RD/RDN, and CDCES, when available. **Grade B** | **X** |  |  |
| 219 | In addition to focused attention on achieving glycemic targets (**Grade A)**, standard preconception care should be augmented with extra focus on nutrition, diabetes education, and screening for diabetes comorbidities and complications. **Grade E** | **X** |  |  |
| 220 | Women with preexisting type 1 or type 2 diabetes who are planning pregnancy or who have become pregnant should be counseled on the risk of development and/or progression of diabetic retinopathy. Dilated eye examinations should occur ideally before pregnancy or in the first trimester, and then patients should be monitored every trimester and for 1 year postpartum as indicated by the degree of retinopathy and as recommended by the eye care provider. **Grade B** | **X** |  |  |
| 221 | Lifestyle behavior change is an essential component of management of GDM and may suffice for the treatment of many women. Insulin should be added if needed to achieve glycemic targets. **Grade A** | **X** |  |  |
| 222 | Insulin is the preferred medication for treating hyperglycemia in GDM. Metformin and glyburide should not be used as first-line agents, as both cross the placenta to the fetus (**Grade A).** Other oral and noninsulin injectable glucose-lowering medications lack long-term safety data. | **X** |  |  |
| 223 | Metformin, when used to treat polycystic ovary syndrome and induce ovulation, should be discontinued by the end of the first trimester. **Grade A** | **X** |  |  |
| 224 | In pregnant patients with diabetes and chronic hypertension, a blood pressure target of 110–135/85 mmHg is suggested in the interest of reducing the risk for accelerated maternal hypertension (**Grade A)** and minimizing impaired fetal growth. **Grade E** | **X** |  |  |
| 225 | Potentially harmful medications in pregnancy (i.e., ACE inhibitors, ARBs, statins) should be stopped at conception and avoided in sexually active women of childbearing age who are not using reliable contraception. **Grade B** | **X** |  |  |
| 226 | Insulin resistance decreases dramatically immediately postpartum, and insulin requirements need to be evaluated and adjusted, as they are often roughly half the pre-pregnancy requirements for the initial few days postpartum. **Grade C** | **X** |  |  |
| 227 | A contraceptive plan should be discussed and implemented with all women with diabetes of reproductive potential. **Grade A** | **X** |  |  |
| 228 | Screen women with a recent history of GDM at 4–12 weeks postpartum, using the 75-g oral glucose tolerance test and clinically appropriate nonpregnancy diagnostic criteria. **Grade B** | **X** |  |  |
| 229 | Women with a history of GDM found to have prediabetes should receive intensive lifestyle interventions and/or metformin to prevent diabetes. **Grade A** | **X** |  |  |
| 230 | Women with a history of GDM should have lifelong screening for the development of type 2 diabetes or prediabetes every 1–3 years. **Grade B** | **X** |  |  |
| 231 | Women with a history of GDM should seek preconception screening for diabetes and preconception care to identify and treat hyperglycemia and prevent congenital malformations. **Grade E** | **X** |  |  |
| 232 | Postpartum care should include psychosocial assessment and support for self-care. **Grade E** | **X** |  |  |
| 234 | Perform an A1C test on all patients with diabetes or hyperglycemia (blood glucose >140 mg/dL [7.8 mmol/L]) admitted to the hospital if not performed in the prior 3 months. **Grade B** |  |  | **X** |
| 235 | Insulin should be administered using validated written or computerized protocols that allow for predefined adjustments in the insulin dosage based on glycemic fluctuations. **Grade B** |  |  | **X** |
| 236 | Insulin therapy should be initiated for treatment of persistent hyperglycemia starting at a threshold ≥180 mg/dL (10.0 mmol/L). Once insulin therapy is started, a target glucose range of 140–180 mg/dL (7.8–10.0 mmol/L) is recommended for the majority of critically ill and noncritically ill patients. **Grade A** |  |  | **X** |
| 237 | More stringent goals, such as 110–140 mg/dL (6.1–7.8 mmol/L), may be appropriate for selected patients if they can be achieved without significant hypoglycemia. **Grade** **C** |  |  | **X** |
| 238 | Basal insulin or a basal plus bolus correction insulin regimen is the preferred treatment for noncritically ill hospitalized patients with poor oral intake or those who are taking nothing by mouth. **Grade A** |  |  | **X** |
| 239 | An insulin regimen with basal, prandial, and correction components is the preferred treatment for noncritically ill hospitalized patients with good nutritional intake. **Grade A** |  |  | **X** |
| 240 | Use of only a sliding-scale insulin regimen in the inpatient hospital setting is strongly discouraged. **Grade A** |  |  | **X** |
| 241 | A hypoglycemia management protocol should be adopted and implemented by each hospital or hospital system. A plan for preventing and treating hypoglycemia should be established for each patient. Episodes of hypoglycemia in the hospital should be documented in the medical record and tracked. **Grade E** |  |  | **X** |
| 242 | The treatment regimen should be reviewed and changed as necessary to prevent further hypoglycemia when a blood glucose value of <70 mg/dL (3.9 mmol/L) is documented. **Grade C** |  |  | **X** |
| 243 | There should be a structured discharge plan tailored to the individual patient with diabetes. **Grade B** |  |  | **X** |

**References**

1. Griffiths Peter. Evidence informing practice: introducing the mini-review. British journal of community nursing. 2002;7:38-9.

2. Party Joanna Briggs Institute Levels of Evidence and Grades of Recommendation Working. Supporting Document for the Joanna Briggs Institute Levels of Evidence and Grades of Recommendation. 2014.

3. Statistics Pakistan Bureau of. Government of Pakistan Bureau of Statistics Karachi. 2021 [June 6th, 2021]; Available from: <https://www.pbs.gov.pk>.

4. Cheema Abdur Rehman, Zaidi Shehla, Najmi Rabia, Khan Fazal Ali, Kori Sultana Ali, Shah Nadir Ali. Availability does not mean utilisation: analysis of a large micro health insurance Programme in Pakistan. Global J Health Sci. 2020;12(10):1-4.

5. Khalid Faraz, Raza Wajeeha, Hotchkiss David R, Soelaeman Rieza H. Health services utilization and out-of-pocket (OOP) expenditures in public and private facilities in Pakistan: an empirical analysis of the 2013–14 OOP health expenditure survey. BMC health services research. 2021;21(1):1-14.

6. Nadeem S., Siddiqi U., Martins R. S., Badini K. Perceptions and Understanding of Diabetes Mellitus Technology in Adults with Type 1 or Type 2 DM: A Pilot Survey from Pakistan. Journal of diabetes science and technology. 2021;15(5):1052-8. Epub 2021/05/08.

7. Unwin N, Whiting D, Gan D, Jacqmain O, Ghyoot G. International Diabetes Federation Diabetes Atlas. IDF Diabetes Atlas. 2009;4.

8. Islam Jessica Yasmine, Zaman Mohammad Mostafa, Bhuiyan Mahfuz Rahman, Haq Syed Atiqul, Ahmed Shamim, Al-Qadir Ahmad Zahid. Prevalence and determinants of hyperglycaemia among adults in Bangladesh: results from a population-based national survey. BMJ open. 2019;9(7):e029674.

9. Meo S. A., Zia I., Bukhari I. A., Arain S. A. Type 2 diabetes mellitus in Pakistan: Current prevalence and future forecast. JPMA The Journal of the Pakistan Medical Association. 2016;66(12):1637-42. Epub 2016/12/08.

10. Federation International Diabetes. IDF Diabetes Atlas. 2021 [21st June 2022]; Available from: <https://diabetesatlas.org/>.

11. Adnan Muhammad, Aasim Muhammad. Prevalence of type 2 diabetes mellitus in adult population of Pakistan: a meta-analysis of prospective cross-sectional surveys. Annals of global health. 2020;86(1).

12. Federation Internasional Diabetes, Atlas ID. International Diabetes Federation. IDF diabetes atlas, 6th edn Brussels, Belgium: International Diabetes Federation. 2013.

13. Kalra Sanjay, Gupta Yashdeep. Primary Care Diabetology. Primary Care. 2015;65(3).

14. Misra Anoop, Ramachandran Ambady, Saboo Banshi, Kesavadev Jothydev, Sosale Arvind, Joshi Shashank, et al. Screening for diabetes in India should be initiated at 25 years age. Diabetes & Metabolic Syndrome: Clinical Research & Reviews. 2021;15(6):102321.

15. Association American Diabetes. Standards of medical care in diabetes—2021 abridged for primary care providers. Clinical diabetes: a publication of the American Diabetes Association. 2021;39(1):14.

16. Shera A. S., Rafique G., Khwaja I. A., Baqai S., Khan I. A., King H. Pakistan National Diabetes Survey prevalence of glucose intolerance and associated factors in North West at Frontier Province (NWFP) of Pakistan. JPMA The Journal of the Pakistan Medical Association. 1999;49(9):206-11. Epub 2000/01/26.

17. Chatterjee Ranee, Venkat Narayan KM, Lipscomb Joseph, Phillips Lawrence S. Screening adults for pre-diabetes and diabetes may be cost-saving. Diabetes care. 2010;33(7):1484-90.

18. Federation International Diabetes. Global Diabetes Plan 2011-2021. 2021 [21st June 2022]; Available from: <https://idf.org/our-activities/advocacy-awareness/resources-and-tools/129-global-diabetes-plan-2011-2021.html>.

19. Shrestha Nipun, Mishra Shiva Raj, Ghimire Saruna, Gyawali Bishal, Mehata Suresh. Burden of diabetes and prediabetes in Nepal: a systematic review and meta-analysis. Diabetes Therapy. 2020;11(9):1935-46.

20. Gaziano Thomas A, Abrahams-Gessel Shafika, Denman Catalina A, Montano Carlos Mendoza, Khanam Masuma, Puoane Thandi, et al. An assessment of community health workers' ability to screen for cardiovascular disease risk with a simple, non-invasive risk assessment instrument in Bangladesh, Guatemala, Mexico, and South Africa: an observational study. The Lancet Global Health. 2015;3(9):e556-e63.

21. Najafi Behzad, Farzadfar Farshad, Ghaderi Hossein, Hadian Mohammad. Cost effectiveness of type 2 diabetes screening: A systematic review. Med J Islam Repub Iran. 2016;30:326-.

22. Hsu Chih-Cheng, Lee Cheng-Hua, Wahlqvist Mark L., Huang Hsiao-Ling, Chang Hsing-Yi, Chen Likwang, et al. Poverty increases type 2 diabetes incidence and inequality of care despite universal health coverage. Diabetes care. 2012;35(11):2286-92. Epub 08/21.

23. Robbins J. M., Vaccarino V., Zhang H., Kasl S. V. Socioeconomic status and diagnosed diabetes incidence. Diabetes research and clinical practice. 2005;68(3):230-6. Epub 2005/06/07.

24. Krishnan Supriya, Cozier Yvette C., Rosenberg Lynn, Palmer Julie R. Socioeconomic status and incidence of type 2 diabetes: results from the Black Women's Health Study. Am J Epidemiol. 2010;171(5):564-70. Epub 02/04.
